# Potential Association Between Atherogenic Coefficient, Prognostic Nutritional Index, and Various Obesity Indices in Diabetic Nephropathy

**DOI:** 10.3390/nu17081339

**Published:** 2025-04-14

**Authors:** Mohamed-Zakaria Assani, Marius Bogdan Novac, Anda Lorena Dijmărescu, Constantin-Cristian Văduva, Ionela Mihaela Vladu, Diana Clenciu, Adina Mitrea, Roxana-Viorela Ahrițculesei, Alexandra-Ștefania Stroe-Ionescu, Alexandru-Dan Assani, Daniel Cosmin Caragea, Mihail Virgil Boldeanu, Isabela Siloși, Lidia Boldeanu

**Affiliations:** 1 Doctoral School, University of Medicine and Pharmacy of Craiova, 200349 Craiova, Romania; mohamed.assani@umfcv.ro (M.-Z.A.); roxana.blendea@gmail.com (R.-V.A.); alexandra.stroe@yahoo.com (A.-Ș.S.-I.); alexandruassani@gmail.com (A.-D.A.); 2 Department of Immunology, Faculty of Medicine, University of Medicine and Pharmacy of Craiova, 200349 Craiova, Romania; isabela_silosi@yahoo.com; 3 Department of Anesthesiology and Intensive Care, Faculty of Medicine, University of Medicine and Pharmacy of Craiova, 200349 Craiova, Romania; mariusnovac2005@yahoo.com; 4 Department of Obstetrics and Gynecology, Faculty of Medicine, University of Medicine and Pharmacy of Craiova, 200349 Craiova, Romania; lorenadijmarescu@yahoo.com (A.L.D.); cristian.vaduva@umfcv.ro (C.-C.V.); 5 Department of Diabetes, Nutrition and Metabolic Diseases, Faculty of Medicine, University of Medicine and Pharmacy of Craiova, 200349 Craiova, Romania; ionela.vladu@umfcv.ro (I.M.V.); dianaclenciu@yahoo.com (D.C.); ada_mitrea@yahoo.com (A.M.); 6 Department of Nephrology, Faculty of Medicine, University of Medicine and Pharmacy of Craiova, 200349 Craiova, Romania; 7 Department of Microbiology, Faculty of Medicine, University of Medicine and Pharmacy of Craiova, 200349 Craiova, Romania; lidia.boldeanu@umfcv.ro

**Keywords:** type 2 diabetes mellitus, diabetic nephropathy, visceral adiposity index, atherogenic coefficient, prognostic nutritional index, chronic kidney disease, metabolic syndrome

## Abstract

**Background/Objectives**: Type 2 diabetes mellitus (T2DM), is a rapidly growing global health concern, often accompanied by chronic kidney disease (CKD) and metabolic disturbances. Obesity-related indices, such as the visceral adiposity index (VAI) and body adiposity index (BAI), have been linked to cardiovascular and renal complications in diabetic patients. However, studies integrating both the atherogenic coefficient (AC) and prognostic nutritional index (PNI) for evaluating diabetic nephropathy (DN) remain limited. This study aimed to assess the associations of obesity-related indices with immunological and nutritional factors in patients with T2DM and prediabetes (PreDM). **Methods**: A retrospective, cross-sectional study was conducted over six months at a university clinical hospital in Dolj County, Romania. The study enrolled 268 newly diagnosed T2DM patients and 150 PreDM patients. Anthropometric parameters, laboratory tests, and demographic data were collected. AC and PNI were calculated using standard formulas, and statistical analyses were performed to determine their associations with metabolic and inflammatory markers. **Results**: Our study found that T2DM patients had significantly lower PNI values, indicating mild malnutrition, while PreDM patients maintained a normal nutritional status. AC was significantly higher in T2DM patients, correlating with lipid profile alterations and systemic inflammation. Obesity indices, particularly VAI, were significantly elevated in T2DM patients with higher AC values. Statistically significant differences in total cholesterol, low-density lipoprotein cholesterol (LDL-c), and triglycerides were observed between AC subgroups, reinforcing its role in cardiovascular risk assessment. **Conclusions**: The findings highlight the potential of AC and PNI as biomarkers for assessing nutritional, inflammatory, and lipemic status in diabetic patients. The significant associations between obesity-related indices, lipid profiles, and inflammation markers suggest that early assessment of these parameters may potentially aid in predicting diabetic complications. Further studies are needed to explore the clinical utility of AC and PNI in managing T2DM and CKD progression. Future research should investigate how the lipidic spectrum alters the progression of DN across various patient groups with diabetes and prediabetes

## 1. Introduction

Diabetes ranks as one of the fastest-growing global health crises of the twenty-first century, as highlighted in the 10th edition of the International Diabetes Federation (IDF) Diabetes Atlas. Estimates suggest that in 2021, 537 million people globally were living with diabetes; this number is expected to increase to 643 million by 2030 and soar to 783 million by 2045. Additionally, it is anticipated that 541 million individuals will experience impaired glucose tolerance by 2021 [1].

Diabetic nephropathy represents a significant global health challenge, serving as a major contributor to the prevalence of CKD and the associated cardiovascular mortality. In 2021, the condition impacted over 107 million individuals worldwide, resulting in more than 2 million fatalities and 11 million disability-adjusted life years (DALYs). The economic ramifications of diabetes reached approximately USD 1.31 trillion in 2015, accounting for around 1.8% of the global gross domestic product (GDP), with a considerable portion of this financial burden stemming from complications such as nephropathy. Proactive management and early intervention strategies are critical in mitigating these adverse effects and improving patient outcomes [2].

Prediabetes (PreDM) is a medical condition that precedes the onset of T2DM, characterized by elevated blood glucose levels that are higher than normal but do not reach the diagnostic threshold for diabetes. This condition is commonly defined as an intermediate state of hyperglycemia, with a significant likelihood of progressing to T2DM [3].

The American Diabetes Association (ADA) defines prediabetes as a fasting plasma glucose (FPG) level between 100 and 125 mg/dL, or glycated hemoglobin A1c (HbA1c) levels varying from 5.7% to 6.4% [3,4,5]. The shift from prediabetes to diabetes may occur slowly over many years, and once diabetes is identified, it cannot be reversed [4].

The visceral adiposity index (VAI), a newly introduced body adiposity measure, serves as an indicator for chronic kidney disease (CKD) [5]. Research has established a significant connection between obesity and prediabetes. Specifically, central (visceral) obesity is closely associated with the onset of T2DM. Additional risk factors encompass physical inactivity, high blood pressure, dyslipidemia (elevated triglycerides or low levels of high-density lipoprotein cholesterol), a family history of diabetes, gestational diabetes, and smoking habits [6]. Obesity is a significant cardiovascular disease (CVD) risk factor and is linked to other major risk factors, such as T2DM, hypertension, and dyslipidemia; these conditions may arise from metabolic abnormalities related to obesity [7].

T2DM accounts for 90% of diabetes cases and typically arises from insulin resistance combined with a relative lack of insulin, leading to elevated glucose levels. Those with T2DM may show no symptoms and are often diagnosed only after experiencing cardiovascular complications. CKD significantly impacts global morbidity and mortality rates [8].

Diabetic nephropathy (DN), recognized as a significant microvascular complication of diabetes, affects approximately 30 to 40 percent of individuals diagnosed with the condition and has emerged as the primary contributor to CKD globally [9]. The prevalence of CKD related to diabetes significantly rises as individuals age, highlighting a concerning trend in which older adults are increasingly affected by complications arising from diabetes [10]. Recent findings indicate that DN is a metabolic disorder closely associated with protein malnutrition, low-grade chronic inflammation, and immune modulation, all of which significantly influence the development and outcomes of the disease [11,12,13,14].

Our research commenced with the objective of evaluating the implications of inflammation and atherosclerosis, as well as the atypical glycemic and lipemic profiles associated with CKD, with T2DM patients being appropriate subjects for this investigation. Supporting this premise is the PREDATORR study conducted in Romania, which examined the prevalence of T2DM and its associations with various risk factors. The PREDATORR study found that the prevalence of DM escalates with age and exhibits a higher incidence in males compared to females. Specifically, the age- and sex-adjusted prevalence of prediabetes was determined to be 16.5% (95% CI 14.8–18.2%), with the highest rates observed in the 60–79 age cohort, particularly among women [15].

Considering the pivotal role of atherosclerosis and inflammation in the advancement of diabetic nephropathy, we aimed to assess these parameters using the atherogenic coefficient (AC) and the prognostic nutritional index (PNI).

Diabetic nephropathy is a major microvascular complication of diabetes mellitus and a leading cause of end-stage renal disease worldwide. It is increasingly recognized that alterations in lipid metabolism play a significant role in its pathogenesis. The atherogenic coefficient (AC), defined as the ratio of non-HDL cholesterol to HDL cholesterol, has emerged as a relevant biomarker reflecting the atherogenic potential of circulating lipids. In patients with diabetic nephropathy, an elevated AC indicates a pro-atherogenic lipid profile that is closely associated with endothelial dysfunction, glomerular injury, and increased cardiovascular morbidity. Understanding the physiopathological implications of the atherogenic coefficient provides important insights into the interplay between dyslipidemia, renal impairment, and cardiovascular risk in diabetic populations [16].

For the analysis of the lipid spectrum, we utilized the AC, which in its formula consists of total cholesterol (TC) and high-density lipoprotein cholesterol (HDL-c). To our knowledge, there are no studies that integrate both PNI and the AC in evaluating DN. AC is referenced in the literature concerning polycystic ovary syndrome, a condition associated with metabolic disturbances such as dyslipidemia, insulin resistance, cardiovascular disease, and an increased risk of T2DM [17]. It is widely recognized that T2DM is associated with a range of metabolic disturbances, including insulin resistance, impaired glucose metabolism, and abnormalities in lipid levels, all of which contribute to the complexity of the disease [1].

Research on how obesity relates to the nutritional and immunological status of Romanian individuals is limited. Consequently, this retrospective study evaluated the link between obesity-related indices and immunological and nutritional factors, including the PNI and AC, in patients with T2DM and PreDM. The study utilized data from a university clinical hospital representing Dolj County, Romania, and aimed to identify potential correlations between these factors.

In recent years, PNI has become a significant prognostic marker for several conditions, such as heart failure, infectious diseases, and T2DM [18]. PNI is assessed through the lymphocyte (LYM) count in peripheral blood and serum albumin (ALB), a biomarker that reflects nutritional status, immune function, and inflammation levels. The concept was initially established by Japanese researchers Onodera et al. [19,20,21,22] who, in 1984, carried out a study to evaluate the nutritional and immune health of cancer patients undergoing gastrointestinal surgery. PNI has been identified as a significant predictor of mortality among elderly patients suffering from CKD, indicating its crucial role in assessing the health risks and outcomes in this vulnerable population [23].

## 2. Materials and Methods

Studies involving interventions with animals or humans, as well as other research necessitating ethical approval, must enumerate the approving authority alongside the relevant ethical approval code. We conducted a non-interventional, cross-sectional epidemiological study for six months. This study involved the enrollment of 268 consecutive patients who were newly diagnosed with T2DM. In parallel, a control group was established, comprising 150 patients with prediabetes who met specific criteria regarding age, gender distribution, and urban versus rural status. The study adhered to the Declaration of Helsinki and received approval from the Ethics Committee of the Filantropia Municipal Clinical Hospital (no. 886/15 January 2024), located in Dolj, Romania.

### 2.1. Selection of Patients, Evaluation of Medical History, Assessment of Biometric Parameters, and Collection of Demographic Data

In order to be qualified to be included in the study, participants had to meet certain conditions: individuals had to be over eighteen years old, they must be diagnosed with prediabetes or type 2 diabetes, while, for the patients with T2DM, another criteria in order to be qualified for the study was the presence of DN. Patients were selected from the Outpatient Diabetes, Nutrition, and Metabolic Diseases Departments of the Filantropia Municipal Clinical Hospital Craiova. All participants enrolled in the study voluntarily, having initially given their informed consent.

We excluded thirty-four patients diagnosed with non-type 2 diabetes mellitus, eighteen patients who presented with autoimmune disease as a comorbidity, and twenty patients for whom critical data, such as waist circumference and body mass index, were lacking; these parameters were deemed essential for the purposes of our study.

Patients diagnosed with chronic microvascular complications of T2DM, such as diabetic peripheral polyneuropathy (eighty-six patients) and diabetic retinopathy (sixty-two patients), were excluded from the study. Diabetic retinopathy (DR) was identified through a dilated fundus examination [24]. Following the guidance of ADA, the assessment of diabetic peripheral neuropathy involved testing temperature sensation (which indicates small fiber function) and vibration sensation (which reflects large fiber function), along with identifying typical symptoms such as pain, dysesthesias, and numbness [24].

The study focused on a cohort of patients who have been newly diagnosed with T2DM and DN. The control cohort was represented by patients diagnosed with PreDM. We evaluated the presence of CKD using the guidelines set by Kidney Disease: Improving Global Outcomes (KDIGO). CKD was characterized by a urinary albumin-to-creatinine ratio (UACR) exceeding 30 mg/g and/or an estimated glomerular filtration rate (eGFR) below 60 mL/min/1.73/m^2^, as outlined in the KDIGO 2021 Guidelines [25]. DKD, also referred to as DN, is characterized by the presence of CKD in conjunction with diabetes. The Chronic Kidney Disease Epidemiology Collaboration (CKD-EPI) equation based on serum creatinine (Scr) levels was employed to calculate eGFR, utilizing the formula provided by MDCalc [26].

In the prediabetic cohort, one hundred patients were excluded from the study. This cohort comprised individuals under the age of eighteen, pregnant women, those who had experienced an acute infection or inflammatory disease within the preceding month, individuals diagnosed with chronic infections or inflammatory disorders, as well as those patients with a history of cancer.

The study sought to gather extensive data on different dimensions of health and lifestyle. This encompassed anthropometric measures, medical variables, laboratory test outcomes, along with demographic and lifestyle details, all obtained through a structured interview questionnaire.

The analysis of demographic variables included age, sex, monthly household income, and educational attainment levels. Additionally, lifestyle and health-related factors were assessed, specifically focusing on historical data regarding tobacco and alcohol use, familial predisposition to hypertension, diabetes mellitus, and cardiovascular diseases. The study also considered the weekly duration of intentional moderate physical activity.

Several patients in the cohort were receiving antihypertensive therapy, specifically with perindopril or amlodipine as well as a combination of perindopril and indapamide. Additionally, statin therapy was utilized, with atorvastatin and rosuvastatin being the agents prescribed.

In terms of dietary management, it was observed that the majority of patients had not received nutrition plans from a specialist. Additionally, post-consultation assessments revealed that participants engaged in physical activity on fewer than four days per week, classifying them as sedentary individuals.

### 2.2. Evaluation of Diabetes and Prediabetes

Prediabetes can be identified based on any of the following criteria: (1) a diagnosis made by a qualified healthcare provider; (2) a hemoglobin A1c (HbA1c) level greater than 5.7% but less than 6.5%; (3) a fasting plasma glucose (FPG) level between 5.6 mmol/L and 7.0 mmol/L; or (4) a 2 h FPG result from an oral glucose tolerance test (OGTT) that is within the range of 7.8 mmol/L to 11.0 mmol/L [27]. Patients with obesity, particularly abdominal or visceral obesity, along with dyslipidemia characterized by elevated triglycerides and/or reduced high-density lipoprotein cholesterol (HDL-c), as well as hypertension, who fulfilled the aforementioned criteria were incorporated into the prediabetes cohort group.

A diabetes diagnosis is established when any of the following criteria are satisfied: a confirmed diagnosis from healthcare providers; an HbA1c level above 6.5%; a fasting plasma glucose (FPG) level of 7.0 mmol/L or higher; a random blood glucose level of 11.1 mmol/L or more; a two-hour post-OGTT blood glucose level exceeding 11.1 mmol/L; or a random glucose measurement accompanied by classic hyperglycemic symptoms, such as polyuria, polydipsia, and unexplained weight loss, or indicators of hyperglycemic crises [27].

Out of 268 patients with T2DM, 48 completed the study, as did 50 out of 150 patients with PreDM, both included in the final analysis.

### 2.3. Assessment of Different Indices Related to Obesity (BMI, WHR, WHtR, BAI, and VAI)

We calculated the body mass index (BMI) from the participants’ height and weight. The formula used is BMI = weight (kilograms)/height^2^ (meters). The patient’s nutritional status was assessed based on BMI, following the WHO criteria [28]. The WHO classifies BMI into three categories: normal weight (18.5–22.9 kg/m^2^), overweight (23.0–25.0 kg/m^2^), and obese (over 25.0 kg/m^2^). We assessed weight using a scale and determined height with a measuring stick attached to the scale.

Measurements of hip circumference (HC) were taken at the femoral trochanters, and waist circumference (WC) was measured at the midpoint between the upper iliac crest and the lower rib cage. The waist-to-hip ratio (WHR), calculated using the formula WC (cm)/HC (cm), serves as a measure of abdominal obesity. Additionally, visceral fat was assessed with the waist-to-height ratio (WHtR), determined by the formula WC (cm)/height (cm). The body adiposity index (BAI) was computed employing the subsequent formula [29]:

BAI = [hip circumference (cm) ÷ height (m)^1.5^] − 18.

The visceral adiposity index (VAI) is determined using the following formula [30]:

$${VAI}_{men}=\left(\frac{WC}{39.68+1.88*BMI}\right)*\left(\frac{TG}{1.03}\right)*\left(\frac{1.31}{HDL\text{-}c}\right)$$

$${VAI}_{women}=\left(\frac{WC}{36.58+1.89*BMI}\right)*\left(\frac{TG}{0.81}\right)*\left(\frac{1.52}{HDL\text{-}c}\right)$$

We divided WHR, WHtR, BAI, and VAI into quarters because standard categories are lacking.

### 2.4. Laboratory Investigations

Once we gathered the anthropometric data, we brought the subjects to the lab for further examination.

We evaluated laboratory data using the chemiluminescence immunological method and an automatic immunoassay analyzer (Cobas e411, Roche Diagnostics GmbH, Mannheim, Germany). The data included HbA1c, fasting plasma glucose (FPG), two-hour plasma glucose after a 75 g oral glucose tolerance test (2hPG), total cholesterol (TC), low-density lipoprotein cholesterol (LDL-c), high-density lipoprotein cholesterol (HDL-c), total triglycerides (TG), blood urea nitrogen (BUN), creatinine (CREA), uric acid (UA), C-reactive protein (CRP), and albumin (ALB).

By employing flow cytometry and Coulter’s principle, we successfully acquired an expanded leukocyte formula based on five parameters (Ruby Cell-Dyne, Abbott, Abbott Park, IL, USA) and identified several hemogram markers: white blood cells/leukocytes (WBC), neutrophils (NEU), lymphocytes (LYM), monocytes (MON), hemoglobin (Hb), platelets (PLT), and hemoglobin (Hb).

Creatinine serum levels were measured, and eGFR was calculated using CKD-EPI formula [26].

### 2.5. Calculations of the Prognostic Nutritional Index and Atherogenic Coefficient Score

PNI is determined using the absolute lymphocyte count and serum ALB level. It is calculated based on the recognized formula as follows [31]:

PNI = albumin level (in grams per liter) + 0.005 × lymphocyte count per microliter.

Interpretation: PNI value ≥ 50—normal, PNI value < 50—mild malnutrition, PNI value < 45—moderate-to-severe malnutrition, PNI value < 40—serious malnutrition.

Given that the mean PNI score for the 50 PreDM patients was 72.69 and for the 48 T2DM patients was 48.94, we categorized the patients into two groups based on their PNI scores. The low PNI group consisted of patients with scores below 72.69 for PreDM and below 48.94 for T2DM, while the high PNI group included those with scores of 72.69 or greater for PreDM and 48.94 or greater for T2DM.

When assessing AC, we utilized the following resources formula [32]:

AC = (TC–HDL-c/HDL-c).

Interpretation: a value of over 3 was classified as abnormal and indicative of high cardiovascular disease [32].

Given that the mean AC score was 2.74 for the 50 PreDM patients and 3.05 for the 48 T2DM patients, we categorized the patients into two groups based on these mean scores: the low AC group (AC < 2.74 for PreDM and AC < 3.05 for T2DM) and the high AC group (AC ≥ 2.74 for PreDM and AC ≥ 3.05 for T2DM).

### 2.6. Statistical Analysis

We processed and managed patient data from medical records using Microsoft Excel. For data analysis, we employed GraphPad Prism 10.3.1 (LLC, San Diego, CA, USA). The D’Agostino and Pearson normality tests were applied to assess the normality of the data.

The means and standard deviations (SD) for the following variables are provided: weight, height, WC, HC, WHtR, BMI, BAI, WBC, NEU, LYM, PLT, Hb, CKD-EPI, ALB, HbA1c, FPG, 2hPG, BUN, creatinine, UA, TC, LDL-c, HDL-c, TG, and BUN, all of which exhibited normal distributions. In contrast, WHR, VAI, CRP, ESR, and MON were found to have non-normal distributions; therefore, these data are presented as the median with interquartile range. The values in this category are represented as percentages.

We evaluated continuous variables with either the one-way ANOVA or the Kruskal–Wallis test, which is suitable for non-Gaussian distributions, to identify differences between the groups. For categorical variables, we employed the χ2 test.

Spearman’s coefficients (−1 < rho < 1) were employed to assess significant correlations between the levels of BMI, height, weight, BAI, VAI, WC, HC, WHR, WHtR, AC, PNI, ALB, CRP, WBC, NEU, LYM, TC, TG, LDL-c, HDL-c, and ESR.

Receiver operating characteristic (ROC) curves were employed to assess the sensitivity and specificity of various parameters, including HbA1c, ALB, FPG, PNI, CKD-EPI, 2hPG, AC, creatinine, VAI, BAI, TC, HDL-c, and LDL-c.

## 3. Results

### 3.1. An In-Depth Exploration of the Clinical and Demographic Profiles of Individuals Diagnosed with Prediabetes and Diabetes

In the present study, we investigated 48 patients newly diagnosed with T2DM, whose ages ranged from 28 to 83 years, with a mean age of 64.25 years and a standard deviation (SD) of 11.95, as presented in Table 1.

This cohort comprised an equitable representation of 24 women and 24 men. In the control cohort, designated as PreDM group, the mean of the age was 48.60 years, with a value of the standard deviation of 7.68; in this group, women accounted for 60% of the participants. A statistically significant age disparity was noted (*p* < 0.0001), whereas no significant difference was observed concerning gender (*χ*^2^(1) = 0.99, *p* = 0.319).

Regarding the patients’ places of residence, it was noted that the majority of individuals in both the T2DM and PreDM groups, consisting of 27 and 34 patients respectively, lived in urban areas.

Furthermore, no meaningful statistical difference was noted between the two groups, concerning their residential status (*χ*^2^(1) = 1.44, *p* = 0.230).

An individual’s medical history serves as a critical distinguishing factor between the T2DM cohort and the PreDM cohort, as substantiated by statistical data. Hypertension, dyslipidemia, and hepatosteatosis were prevalent in over 68% of the patients, indicating significant comorbidity within this population. Furthermore, the T2DM cohort displayed statistically elevated mean values for both systolic blood pressure (SBP) and diastolic blood pressure (DBP).

The analysis of anthropometric parameters, including height, weight, WC, and HC, alongside a range of obesity-related indices such as BAI, BMI, WHR, and, WHtR, revealed statistically significant differences in mean weight between the two groups (*p* = 0.047). Additionally, the mean values of BMI within the obese category achieved statistical significance (*p* = 0.01). It is also evident that VAI demonstrated statistical significance between the PreDM group and T2DM group (*p* = 0.04). Furthermore, AC exhibited statistical significance (*p* = 0.001).

The examination of laboratory parameters indicated that the values established for diagnosing T2DM were statistically significantly higher than those of PreDM group, as illustrated in Table 1 (*p* < 0.0001).

In the course of our investigation, a comprehensive analysis of nutritional status and systemic inflammation was conducted. Significant discrepancies were identified in the levels of CRP [20.70 (3.20–76) vs. 0.49 (0.05–162.1), *p* < 0.0001] and ALB (3.82 ± 0.27 vs. 6.19 ± 0.51, *p* < 0.0001) between two distinct groups, specifically individuals diagnosed with T2DM and those classified with PreDM. Furthermore, PNI, which is determined by the count of LYM present in peripheral blood and serum ALB levels, revealed statistically significant differences between the PreDM and T2DM cohorts. Additionally, it was noted that patients diagnosed with T2DM exhibited a condition of mild malnutrition (*p* < 0.0001). Based on the PNI value, which evaluates both inflammatory and nutritional statuses through ALB and CRP levels, it was established that 65% of T2DM patients demonstrated mild malnutrition. Conversely, all PreDM patients were found to maintain a normal nutritional status.

### 3.2. Comparative Analysis of Clinical Characteristics Among the Atherogenic Coefficient AC in People with Pre-Diabetes and Type 2 Diabetes Mellitus

The two groups were subsequently divided into two subgroups based on the criteria of AC: low AC, which is defined as values less than 2.74 and 3.05, respectively, and high AC, characterized by values that are greater than or equal to 2.74 and 3.05, respectively, as outlined in Table 2 and Table 3.

The cohort classified as PreDM revealed a noteworthy proportion of patients, specifically 62%, who exhibited an AC measurement of less than 2.74. In the subgroup designated as T2DM, 60% of the individuals within this specific group demonstrated an AC measurement of less than 3.05.

There were no statistically significant differences in age and area of residence (*p* ≥ 0.05) among both prediabetes subgroups (AC < 2.74 and AC ≥ 2.74), as well as within the T2DM subgroups (AC < 3.05 and AC ≥ 3.05). Nonetheless, a significant difference was noted in gender between the T2DM subgroups (AC < 3.05 and AC ≥ 3.05) (*p* = 0.038).

In the PreDM group, we observed statistical differences in medical histories and clinical conditions, particularly regarding alcohol consumption and hypertension (*p* = 0.005 and *p* = 0.41, respectively). Regarding anthropometric measurements, waist circumference (*p* = 0.042) was significantly higher in individuals with an abdominal circumference (AC) of ≥2.74, while the waist-to-height ratio neared significance (*p* = 0.056). Additionally, the VAI reached statistical significance (*p* = 0.036).

The lipid profile analysis revealed significantly high TC and LDLc levels in the subgroup with an AC ≥ 2.74 within PreDM group, exhibiting *p*-values of 0.002 and 0.001, respectively. In contrast, HDL-c levels were found to be lower for this subgroup. Moreover, BUN neared significance with a *p*-value of 0.057.

In the subgroups where AC is less than 2.74, significant differences in mean values were observed for WBC (*p* = 0.033), NEU (*p* = 0.023), and LYM (*p* < 0.0001), when compared to those documented in the subgroup with an AC value equal to or greater than 2.74. Furthermore, it was noted that the CRP approached significance with a statistical *p*-value of 0.052.

Regarding the atherogenic coefficient, a significant difference in the mean (*p* < 0.0001) was observed between the two subgroups within the pre-diabetes cohort.

Obesity indices, specifically the waist-to-hip ratio (*p* = 0.047), waist-to-height ratio (*p* = 0.040), and BAI (*p* = 0.039), demonstrated a higher mean for the subgroup exhibiting an AC of 3.05 or greater. We can observe that VAI showed a significantly elevated value for the subgroup with an AC ≥ 3.05 or greater, accompanied by a statistical *p*-value of 0.002.

The lipid profile showed significantly elevated levels of TC (*p* < 0.0001), LDLc (*p* < 0.0001), and TG (*p* = 0.002). In contrast, HDL-c level was significantly lower in the subgroup with an AC ≥ 3.05 (*p* = 0.025) within the T2DM group.

Our observations revealed a substantial difference in renal function, as indicated by a decreased eGFR in the subgroup with an AC of ≥3.05. The analysis conducted by CKD-EPI resulted in a *p*-value of 0.039, whereas a higher level of uric acid produced a *p*-value of 0.023. Concurrently, the glycemic profile demonstrated statistical significance with a *p*-value of 0.048.

In the subgroups where the AC value is below 3.05, the mean values for WBC (*p* = 0.035), NEU (*p* = 0.047), and CRP (*p* = 0.023) demonstrated statistically significant differences when compared to the subgroup with AC equal to or exceeding 3.05. Additionally, it was observed that hemoglobin (*p* = 0.057) and the erythrocyte sedimentation rate (*p* = 0.059) neared the threshold of statistical significance.

Concerning the atherogenic coefficient, a significant difference in the mean was observed (*p* < 0.0001) between the two subgroups within T2DM group.

### 3.3. Comparative Analysis of Clinical Characteristics Among the Prognostic Nutritional Index PNI in Individuals with Pre-Diabetes and Type 2 Diabetes Mellitus

Utilizing PNI cut-off values of 72.69 and 48.94, the two groups were systematically categorized into two distinct subgroups: low PNI (defined as less than 72.69 and less than 48.94, respectively) and high PNI (defined as greater than or equal to 72.69 and greater than or equal to 48.94, respectively), as illustrated in Table 2 and Table 3 above.

The cohort of pre-diabetic individuals indicated that a substantial percentage of patients (52%) displayed a PNI that was equal to or surpassed 72.69. In a similar manner, within the T2DM group, 52% of patients exhibited a PNI that fell below 48.94.

No statistically significant differences were found regarding age, gender, and area of residence (*p* ≥ 0.05) within either pre-diabetes subgroups (PNI < 72.69 and PNI ≥ 72.69) or the type 2 diabetes subgroups (PNI < 48.94 and PNI ≥ 48.94).

In the pre-diabetes mellitus group, a significant link was found (*p* = 0.002) between PNI values below 72.69 and various lifestyle factors, including alcohol intake. Additionally, there was a notable association with primary risk factors for T2DM such as hypertension (*p* = 0.047) and dyslipidemia, which neared statistical significance (*p* = 0.054).

Furthermore, the subgroup with a PNI below 72.69 exhibited increases in body weight and hip circumference (*p* = 0.007 and *p* = 0.048, respectively). Additionally, BMI and HDL-c demonstrated statistically significant differences (*p* = 0.013 and *p* = 0.048, respectively) between the two prediabetic subgroups, after adjusting for the PNI. This nutritional and inflammatory condition is supported by the mean levels of ALB, which displayed significantly lower values (*p* < 0.0001).

In the subgroups with a PNI lower than 72.69, the average values for HBG (*p* = 0.001), WBC (*p* = 0.001), neutrophils NEU (*p* = 0.025), LYM (*p* = 0.0004), and MON (*p* = 0.025) differed significantly from those in the subgroup with a PNI of 72.69 or higher.

Anthropometric parameters, including weight (*p* = 0.045), HC (*p* = 0.046), and BMI —notably in the overweight and obese categories—(*p* = 0.017 and *p* = 0.047, respectively) revealed significantly elevated median values in subgroups with a PNI below 48.94. Additionally, the diabetic subgroup with a PNI under 48.94 exhibited a significantly higher median VAI (*p* = 0.08).

In patients diagnosed with T2DM, individuals classified within the subgroups with a PNI of less than 48.94 demonstrated a mild nutritional and inflammatory status. This group exhibited significantly altered mean values for white blood cells (WBC) (*p* = 0.023), LYM (*p* < 0.0001), and ALB (*p* = 0.0004) in comparison to those within the PNI subgroups of 48.94 or greater.

Patients classified within the PNI < 48.94 subgroup demonstrated mean values for 2hPG that exhibited statistical significance with a *p*-value of 0.049, HbA1c at *p* = 0.021, TC at *p* = 0.009, LDL-c at *p* = 0.035, and TG at *p* = 0.003. These values were significantly different from those documented in the PNI ≥ 48.94 subgroup. Moreover, a statistically significant difference in hemoglobin levels was observed, with a *p*-value of 0.011.

### 3.4. Connections of AC with BAI, VAI, WHR, WHtR, and BMI in the PreDM and T2DM Groups

**Figure 1:**
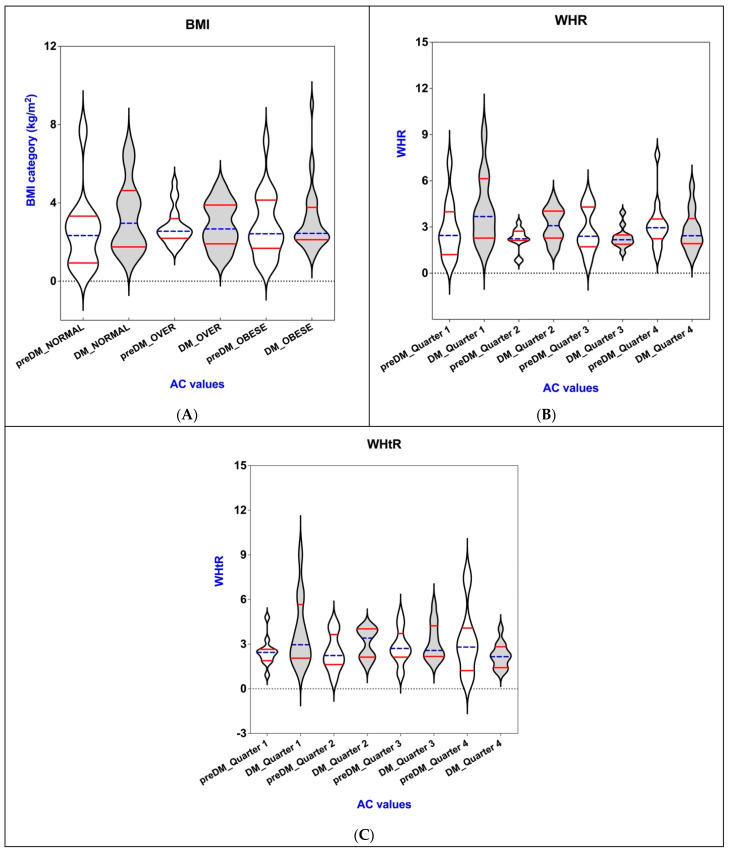

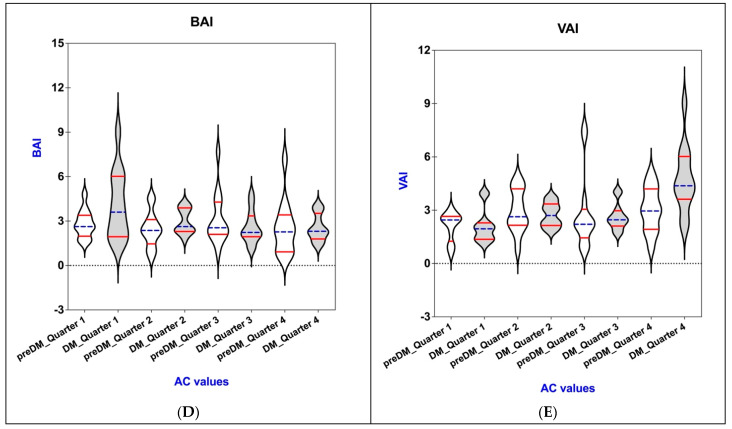
The AC levels for patients with prediabetes (represented in white) or diabetes (indicated in gray) differ across various quarters of obesity-related indices: (**A**) BMI; (**B**) WHR; (**C**) WHtR; (**D**) BAI; (**E**) VAI. The violin plot illustrates the distribution of these indices, with the horizontal blue lines that indicate the median values, while the quartiles are represented by the horizontal red lines.

Table 4 and corresponding Figure 1 illustrate the inter-relationships among the following indices: BAI, VAI, WHR, WHtR, and BMI within the cohorts of individuals diagnosed with PreDM and T2DM.

BMI is classified into three groups: normal weight (18.5–22.9 kg/m^2^), overweight (23.0–25.0 kg/m^2^), and obesity (greater than 25.0 kg/m^2^), based on standards set by the WHO.

In light of the absence of standardized categories, we have systematically categorized the WHR, WHtR, BAI, and VAI into quartiles.

Utilizing ANOVA, we observed that patients with prediabetes were close to reaching statistical significance regarding the quartiles of the waist-to-height ratio, with a *p*-value of 0.054. In contrast, the findings from the Kruskal–Wallis test indicated that prediabetic individuals exhibited statistically significant differences in quartile values for both the WHR with a *p*-value of 0.039, and the VAI, which had a *p*-value of 0.037.

The results from the repeated measures ANOVA revealed a statistically significant difference in WHtR, with a *p*-value of 0.049. Likewise, the BAI showed a significant difference characterized by a *p*-value of 0.033. The Kruskal–Wallis test further indicated that WHR had a *p*-value of 0.042, while VAI exhibited an exceptionally significant *p*-value of less than 0.0001, reinforcing the relevance of these adiposity measures in the analyzed cohort.

The analysis revealed that there were no significant variations in WHR, WHtR, and BAI values across the different quarters within T2DM group. Similarly, when looking at the PreDM group, no statistically significant differences were identified among the various categories of BMI or between the quarterly measurements of BAI. This indicates a consistent pattern in body measurement indices across the studied periods and groups.

### 3.5. Connections of PNI with BAI, VAI, WHR, WHtR, and BMI in the PreDM and T2DM Groups

**Figure 2:**
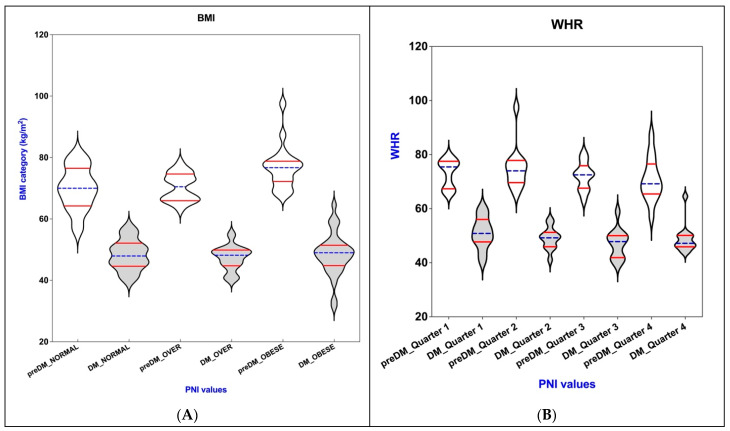

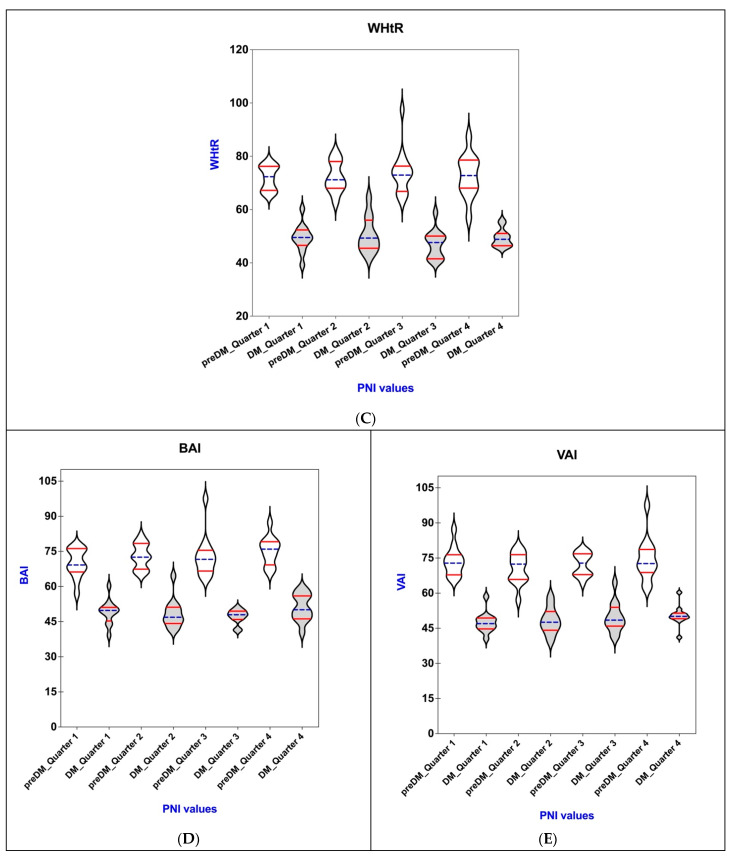
The PNI levels for patients with prediabetes (represented in white) or diabetes (indicated in gray) differ across various quarters of obesity-related indices: (**A**) BMI; (**B**) WHR; (**C**) WHtR; (**D**) BAI; (**E**) VAI. The violin plot illustrates the distribution of these indices, with the horizontal blue lines that indicate the median values, while the quartiles are represented by the horizontal red lines.

Table 5 and Figure 2 illustrate the relationships between PNI, BMI, WHR, WHtR, BAI, and VAI within the PreDM and T2DM cohorts.

BMI is classified by the WHO into normal weight (18.5–22.9 kg/m^2^), overweight (23.0–25.0 kg/m^2^), and obese (over 25.0 kg/m^2^).

In light of the absence of standardized classifications for WHR, WHtR, BAI, and VAI, these metrics have also been stratified into quartiles for analytical purposes.

Utilizing the ANOVA test, we found that patients with a diagnosis of prediabetes exhibited statistically significant differences in BMI values across various categories (*p* = 0.002). Conversely, the Kruskal–Wallis test indicated that the differences between the quartiles of the VAI in prediabetic patients approached the threshold of significance (*p* = 0.052).

In the DM group, the application of the ANOVA test revealed no statistically significant differences in BAI (*p* = 0.34). In contrast, the results from the Kruskal–Wallis test indicated that VAI was nearing statistical significance.

No noteworthy differences were found in the measurements of WHR, WHtR, and BAI when comparing the various quarters within the group of individuals diagnosed with T2DM. This indicates a consistent trend in body composition indices across the different time periods studied in this particular patient population.

In our analysis, we found that there were no statistically significant differences in the values when comparing various (BMI) categories among the participants. Furthermore, when examining the quarterly measurements for both WHR and WHtR, no notable differences emerged within the cohort of individuals who had been diagnosed with T2DM. This indicates that, despite the expectations of variations due to different BMI classifications or changes over time in body composition metrics such as WHR and WHtR, the data remained consistent without meaningful divergence across these measurements within the studied group.

### 3.6. Correlations Between AC, Obesity-Related Indices, and Lipemic Profile in the PreDM and T2DM Groups

**Figure 3:**
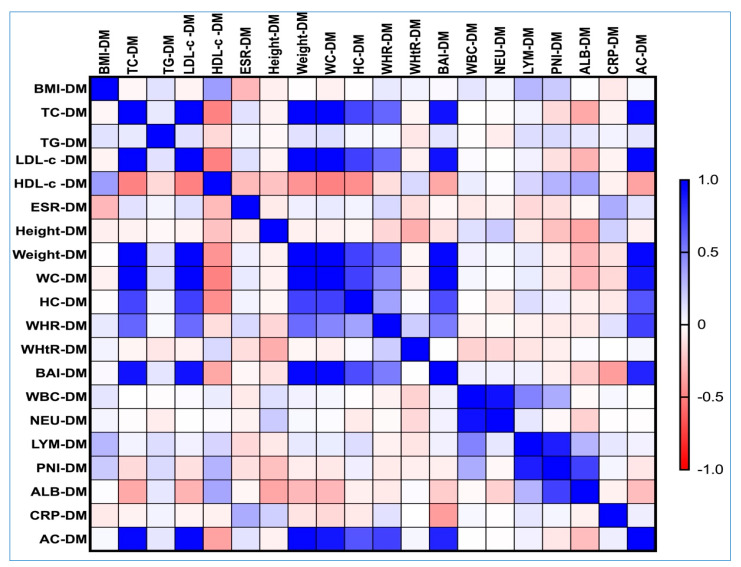
Correlation matrix between AC, lipid spectrum, and obesity-related indices in the T2DM cohort. The correlation heatmap illustrates the relationships between the measured indicators. Strong positive correlations are represented by bright blue, while strong negative correlations are depicted in bright red.

Spearman’s correlation analysis revealed that the values of AC displayed a significantly stronger association with various obesity-related indices in the T2DM group. This indicates that as AC measurements increased, there was a notable correlation with other obesity indicators, suggesting that AC may serve as an important metric for assessing obesity in patients with T2DM. For detailed visual representation, please refer to Figure 3.

In our study involving individuals with T2DM, we found a strong and statistically significant positive correlation between AC and several parameters. Specifically, we observed correlations with weight (rho = 0.951, *p*-value < 0.0001), WC (rho = 0.892, *p*-value = 0.012), BAI (rho = 0.845, *p*-value = 0.014), TC (rho = 0.950, *p*-value < 0.0001), and LDL-c (rho = 0.953, *p*-value < 0.0001).

A moderate and statistically significant positive correlation was found between HC and the variables in question, with a correlation coefficient (rho) of 0.661 and a *p*-value of 0.031. This indicates that as one variable increases, there is a tendency for hip circumference to increase as well.

Additionally, a strong correlation was observed with WHR, which resulted in a correlation coefficient of 0.740 and a *p*-value of 0.027. This also suggests that changes in the variables analyzed are associated with variations in the waist-to-hip ratio, further underscoring the relationship between these anthropometric measures.

In our analysis, we observed that the levels of AC demonstrated a positive but relatively weak correlation with TG, as indicated by a correlation coefficient (rho) of 0.101 and a *p*-value of 0.049. This correlation is statistically significant, suggesting a potential relationship between these two variables. Similarly, AC also showed a weak positive correlation with ESR, with a rho value of 0.112 and a *p*-value of 0.044, further supporting the idea of a noteworthy albeit modest association.

On the other hand, we found a noteworthy single negative correlation between AC and HDL-c. This relationship exhibited a correlation coefficient of −0.372, with a *p*-value of 0.048, indicating that this negative association is statistically significant as well.

Overall, these findings provide insight into the complex interplay between AC and various lipid and inflammatory markers.

**Figure 4:**
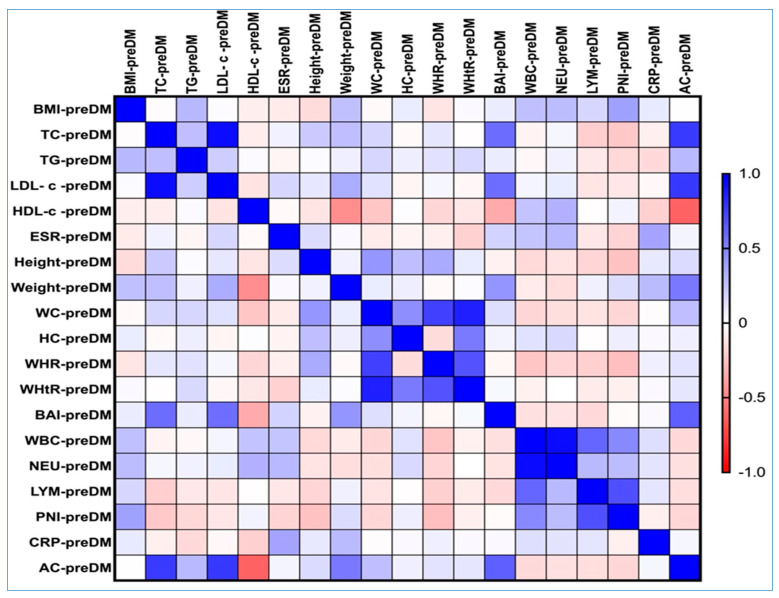
Correlation matrix of AC, lipid spectrum, and obesity-related indices in the PreDM cohort. The correlation heatmap illustrates the relationships between the measured indicators. Strong positive correlations are represented by bright blue, while strong negative correlations are depicted in bright red.

In the PreDM group, our research findings, as depicted in Figure 4, revealed a moderate and statistically significant positive correlation between AC and several key health metrics. Specifically, there was a correlation with body weight (rho = 0.529, *p*-value = 0.047), TC levels (rho = 0.759, *p*-value = 0.026), LDL-c (rho = 0.767, *p*-value = 0.022), and BAI (rho = 0.625, *p*-value = 0.34).

In addition to these notable correlations, the AC values displayed a weaker positive relationship with TG (rho = 0.277, *p*-value = 0.05), height (rho = 0.143, *p*-value = 0.053), WC (rho = 0.255, *p*-value = 0.057), and WHR (rho = 0.111, *p*-value = 0.054).

Interestingly, we also identified a significant negative correlation between AC and HDL-c, where higher abdominal circumference was associated with lower HDL-c levels (rho = −0.617, *p*-value = 0.041). This suggests that as abdominal circumference increases, the levels of this beneficial cholesterol tend to decrease.

### 3.7. Comparative Analysis of Clinical Features Between Subgroups of Females and Males in Pre-Diabetes and Type 2 Diabetes Mellitus Groups

As illustrated in Table 6, we also categorized the PreDM and DM groups according to gender, distinguishing between female and male.

Regarding the PreDM group, it is noteworthy that variables such as BMI for the normal weight category (*p* = 0.021), weight (*p* = 0.049), VAI (*p* < 0.0001), and TG (*p* = 0.050) attained statistical significance.

In the DM group, we observed that the parameters of age (*p* = 0.0003), weight (*p* = 0.019), height (*p* < 0.0001), VAI (*p* = 0.039), BAI (*p* = 0.009), and LDL-c (*p* = 0.045) attained statistical significance.

One of the most significant clinical features that exhibited statistical significance is represented by eGFR, calculated using the CKD-EPI equation for both PreDM and T2DM groups (*p* = 0.039, *p* < 0.0001, respectively). This finding indicates a more pronounced impairment of renal function within the female cohort in both groups.

Regarding the PreDM group, it is noteworthy that variables such as BMI for the normal weight category (*p* = 0.021), weight (*p* = 0.049), VAI (*p* < 0.0001), and TG (*p* = 0.050) attained statistical significance.

### 3.8. Diagnostic Accuracy of Different Indexes and Biomarkers

Our study focused on evaluating the ability of various clinical parameters to differentiate between inflammatory and atherosclerotic conditions in patients with PreDM and T2DM. We conducted a thorough analysis using ROC curve methodology to assess each parameter’s diagnostic performance. The parameters investigated included HbA1c, ALB, FPG, PNI, CKD-EPI, 2 h post-glucose 2hPG, AC, creatinine, VAI, BAI, HDL-c, LDL-c, TC.

**Figure 5:**
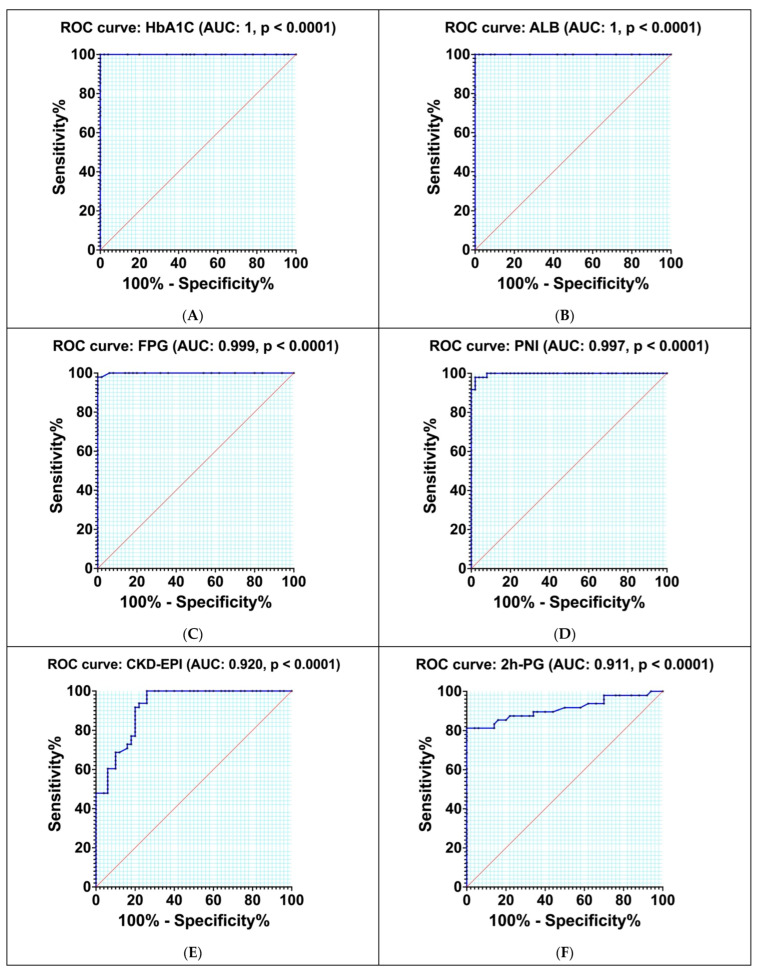

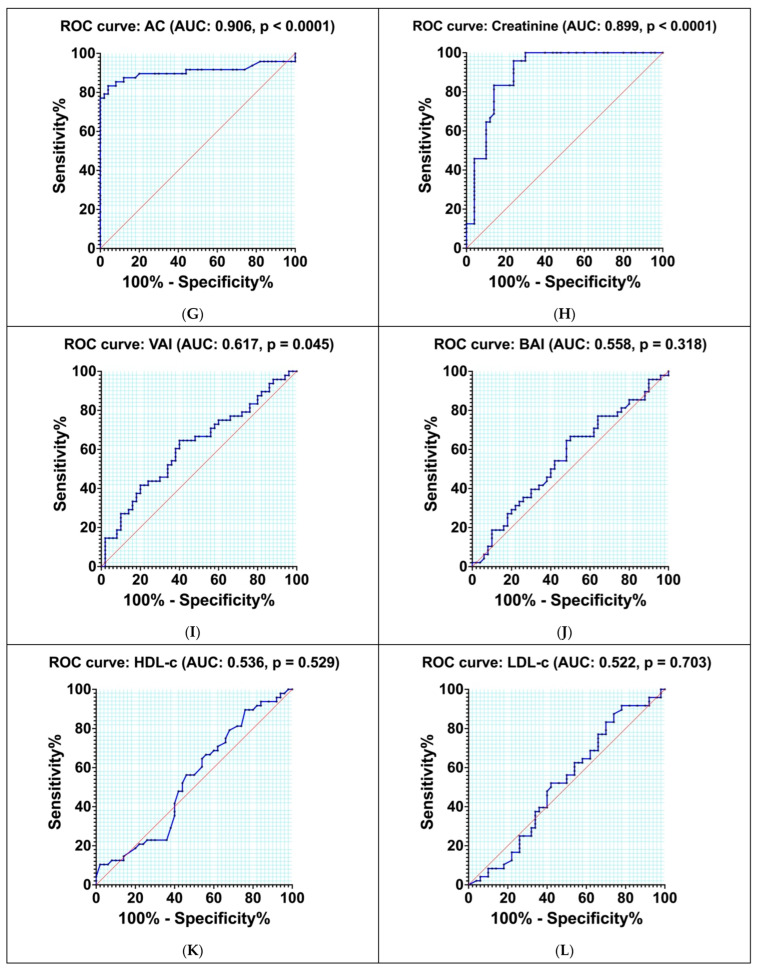

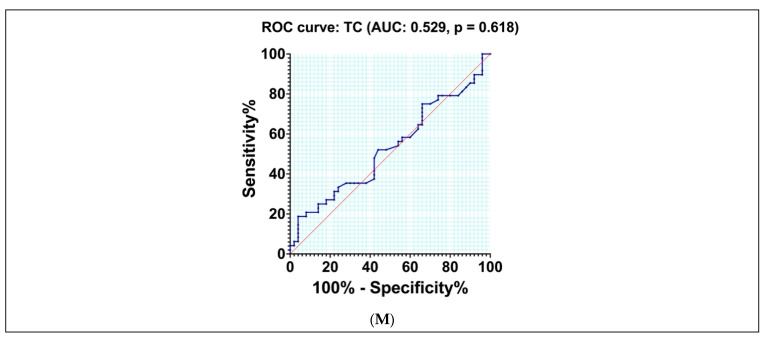
Receiver operating characteristic (ROC) curve for HbA1c (**A**), ALB (**B**), FPG (**C**), PNI (**D**), CKD-EPI (**E**), 2hPG (**F**), AC (**G**), Creatinine (**H**), VAI (**I**), BAI (**J**), HDL-c (**K**), LDL-c (**L**) and TC (**M**).

For each parameter, we established a cut-off value designed to optimize the balance between sensitivity and specificity, thus facilitating effective differentiation between the two medical conditions under study. The results of our analysis, including the ROC curves for each parameter, are presented in Table 7 and illustrated in Figure 5A through M. Analyzing the area under the ROC curve (AUC) and the evaluated parameters, we observed that the highest diagnostic accuracy was achieved with HbA1c (100%) and ALB (100%), followed by FPG (99.90%) and PNI (99.70%). In the case of HDL-c (53.60%), LDL-c (52.20%), and TC (52.90%), our study revealed a much lower diagnostic accuracy.

We selected cut-off levels for each of the examined inflammatory markers based on the sensitivity and specificity of the ROC curves in identifying the inflammatory and atherosclerotic status. AC had the a specificity of 98.00% (sensitivity equal to 79.17%, the Youden index of 0.770, the cut-off value 3.90). PNI presented 97.92% sensitivity and 98.00% specificity; the cut-off value was determined to be 61.45; the Youden index was 0.960.

In our study on anthropometric indices for obesity, the VAI showed a sensitivity of 64.58% and a specificity of 60.00% at a cut-off value of 3.96, resulting in an AUC of 61.70%. This indicates moderate diagnostic accuracy. Similarly, the BAI had a sensitivity of 66.67% and a specificity of 50% at a cut-off value of 30.45, with an AUC of 55.80%. These results highlight the varying effectiveness of these indices in identifying obesity.

## 4. Discussion

By the year 2025, it is projected that approximately 26.6 million individuals globally will be diagnosed with diabetes, culminating in a total of 579.9 million people affected by this disease [33]. WHO forecasts that the increasing prevalence of diabetes will result in it becoming the seventh-leading cause of death worldwide by the year 2030 [34]. Additionally, the population diagnosed with prediabetes—a state characterized by elevated blood glucose levels that do not yet reach the threshold for type 2 diabetes—is anticipated to rise [3].

DR, DKD, and diabetic peripheral neuropathy constitute the most common microvascular complications associated the T2DM [35]. DKD develops gradually and does not present unique clinical symptoms, contributing to 20–40% of T2DM cases [35].

Forecasts suggest that by the year 2030, the global population of individuals diagnosed with prediabetes is expected to exceed 470 million [36]. It is essential to recognize the significance of individuals with PreDM. Individuals diagnosed with prediabetes demonstrate an increased susceptibility to cardiovascular diseases and diabetic microangiopathy relative to those with euglycemic glucose metabolism [37,38].

In this study, we enrolled forty-eight patients with recent diagnoses of T2DM and fifty individuals with PreDM. Our objective was to investigate the correlations between obesity-related metrics and a range of immunological and nutritional parameters, specifically focusing on AC and PNI. Through this analysis, we aimed to elucidate potential associations among these variables.

In our observational study, we identified four noteworthy findings. Notably, 22% of patients with PreDM and 12.5% of those with T2DM were classified as being of normal weight. In contrast, a significant proportion—38% of prediabetic patients and 62.5% of those with T2DM—were categorized as patients with obesity. The second significant observation revealed that the PNI in patients with T2DM was markedly lower compared to those with PreDM. Specifically, T2DM patients exhibited an average PNI of 48.94, indicative of a mild malnutrition state. In contrast, PreDM patients demonstrated a median PNI of 72.69, reflecting a normal nutritional status. Thirdly, it was noted that PreDM patients presenting a PNI value below 72.69 and T2DM patients with a PNI below 48.94 were associated with significantly higher mean values of HC. Finally, within the T2DM cohort, a strong and statistically significant positive correlation was identified between AC and WC, BAI, TC, and LDL-c. Furthermore, the AC measurements demonstrated a weak negative yet statistically significant correlation with HDL-c across both PreDM and T2DM groups, while exhibiting a moderate positive correlation, which was both statistically significant, with TC, LDL-c, and BAI weight.

Obesity and type 2 diabetes are intricately linked through various shared pathophysiological pathways. As a result, patients with obesity are at an increased risk for the development of insulin resistance, dyslipidemia, non-alcoholic fatty liver disease, and various other metabolic syndromes [39,40]. Metabolic syndrome (MetS) exhibits a strong correlation with lipid abnormalities, which are integral to its diagnostic criteria. In Romania, the prevalence of MetS stands at 38.5%. This prevalence exhibits a positive correlation with age and is notably higher among male participants compared to their female counterparts [41]. Obesity causes ongoing, widespread inflammation [42]. Analogous with endometriosis, the development of cellular atypia or malignant transformation can occur as a consequence of a pro-inflammatory microenvironment, primarily influenced by inflammatory cell activity. This milieu promotes neovascularization and facilitates mutations in tumor suppressor genes or oncoproteins, leading to enhanced cellular proliferation and tumorigenesis [43].

Weight management is usually recommended in the treatment of diabetes and various systemic disorders to reduce complication rates [44]. Individuals classified as overweight, even when their BMI does not meet the criteria for obesity, can still exhibit abdominal obesity. This condition, commonly assessed through indices such as the WHR, BAI, or visceral adiposity index, serves as an independent predictor for the onset of hypertension and elevated fasting glucose levels [45,46]. BMI is often criticized for its limited effectiveness in accurately reflecting adiposity. As De Lorenzo has illustrated, a more precise evaluation of body fat can be achieved through the anthropometric assessment of body fat percentage, which provides a clearer understanding of an individual’s body composition [47]. A study from 2020 investigating the adult population in the United States revealed that approximately two-thirds of the participants were classified as either patients with obesity or patients being overweight, indicating a significant prevalence of elevated BMI levels within this demographic [48].

In our study of patients, we noted similar results: 25% of T2DM patients were overweight, and 62.5% were classified as patients with obesity. This trend can be attributed to Romania’s rapid adoption of a relatively high socioeconomic status, which has coincided with rising per capita incomes and contributed to an increasing prevalence of obesity.

Li et al. [29] determined that the three obesity-related indices—BMI, WHtR, and BAI—had a negative correlation with the onset of DR. The authors suggest that using the BMI may not effectively distinguish between general obesity and centripetal obesity, and these two types may influence diabetes development differently [47]. Regarding this aspect, Man et al. [49] identified a positive link between centripetal obesity, characterized by an increased WHR, and its progression to diabetes. In a separate investigation, it was demonstrated that WHR should be regarded as a critical measure for the assessment of centripetal obesity. Moreover, it has been demonstrated that BAI shows a robust linear correlation with the percentage of body fat. Studies suggest a noteworthy link between abdominal obesity and various metabolic risks may be a more significant contributor to diabetic retinopathy than generalized obesity [50]. Although DR differs from DN, both conditions are classified under the category of microvascular complications associated with T2DM [51]. Consequently, the same principles may be relevant for diabetic nephropathy.

In our study, we examined patients exhibiting centripetal obesity associated with T2DM, as evidenced by elevated WHR and WHtR values. Furthermore, approximately 40% of these patients, who had an AC of 3.05 or greater, demonstrated a significant correlation with centripetal obesity.

To the best of our knowledge, there have not been any population-based studies documenting the correlation between BAI, VAI, WHR, or WHtR measures and the atherogenic coefficient in patients recently diagnosed with T2DM or PreDM.

Recent studies highlighted the PNI as a promising clinical biomarker with significant implications for both diagnosis and prognosis in patients with diabetes who are experiencing microvascular complications [9,51,52,53,54,55].

Aktas et al. found that the PNI in patients with T2DM was significantly lower compared to that of healthy controls. [51]. Furthermore, it was observed that the PNI of the diabetic subjects experiencing microvascular complications, including diabetic nephropathy, diabetic retinopathy, and diabetic neuropathy, was significantly lower than that of the healthy control group and the diabetic patients who did not present with microvascular complications. The concept has been substantiated by our research regarding diabetic nephropathy.

Our study yielded analogous results involving newly diagnosed patients with T2DM. The PNI of T2DM patients was significantly lower than that of patients diagnosed with PreDM. Furthermore, the PNI indicated that individuals with T2DM exhibited a mild state of malnutrition, whereas those with PreDM presented with a normal nutritional status at the time of diagnosis.

Zhang et al. postulated that the PNI, which integrates the domains of nutrition, immunology, and inflammation—critical factors in DN—may provide a more reliable prognostic indicator for end-stage renal disease (ESRD) in DN patients compared to traditional markers such as serum albumin levels, inflammatory indices, or lymphocyte counts [9]. In a subsequent investigation, the same author identified a robust correlation between elevated psychological distress PNI and both mortality risk and the incidence of CKD among participants [52].

Our investigation revealed that within the T2DM cohort, there exists a moderate and statistically significant negative correlation between the PNI and various anthropometric and metabolic parameters, specifically weight, WC, HC, WHtR, BAI, and FPG. Additionally, PNI demonstrated a weak inverse correlation with BMI and HbA1c. Interestingly, PNI exhibited a singular positive correlation, though weak yet statistically significant, with CKD-EPI values. These findings corroborate previous research in this area. Our findings were in agreement with those mentioned in other studies. PNI was found to be positively connected with eGFR and negatively correlated with BMI, HbA1c, and fasting blood glucose, according to Aktas et al. [51].

Additionally, Zhang et al. [9,52], in their study, revealed a positive correlation between the PNI and eGFR. Conversely, in the study, the PNI also exhibited negative correlations with several key parameters, including red cell distribution width, HbA1c, urine albumin-to-creatinine ratio, neutrophil-to-lymphocyte ratio, markers of glomerular injury, and high-sensitivity C-reactive protein.

The PNI consists of serum albumin and blood lymphocyte count, both readily accessible in most medical laboratories. As a result, the PNI serves as an easily measurable and cost-effective marker since analyzing serum albumin and lymphocyte counts is inexpensive.

Dyslipidemia constitutes a prevalent risk factor for cardiovascular disease, which, in turn, serves as the primary cause of morbidity and mortality among patients with CKD and T2DM. Furthermore, obesity and metabolic syndrome are intrinsically linked to DN, the leading cause of chronic kidney disease globally [56]. Patients with DN exhibit elevated levels of cholesterol, triglycerides, and apolipoprotein B (ApoB)-associated lipoproteins, including very-low-density lipoprotein (VLDL), intermediate-density lipoprotein (IDL), LDL, and lipoprotein(a) (Lpa). Concurrently, they show reduced HDL [57,58]. Our research additionally indicated that the diabetic cohort exhibited elevated levels within the lipidic spectrum. This group displayed a lipid profile characterized by significantly increased levels of TC (*p* < 0.0001), LDLc (*p* < 0.0001), and TG (*p* = 0.002). In contrast, the HDL-c level was markedly reduced in the subgroup presenting an AC ≥ 3.05 (*p* = 0.025) within T2DM population.

The atherogenic coefficient serves as an indicator of the atherogenic potential across the complete range of lipoprotein fractions, with the atherogenic coefficient being notably elevated in the cases group, for Yang et al.’s study [59]. In the current study, patients with T2DM demonstrating an AC of 3.05 or greater exhibited a statistically significant positive correlation with HbA1C, as evidenced by a *p*-value of 0.048. Our findings are in agreement with those mentioned in Sahani et al., as presented in their study of the positive correlation of atherogenic coefficient and glycemic status (HbA1C) [60].

Cardiovascular disease is the primary comorbidity associated with CKD, and a significant number of CKD patients succumb to cardiovascular issues prior to reaching kidney failure. Identifying anatomical and functional cardiovascular abnormalities, along with hypertension, is crucial for effectively managing these complications [61]. Thus, a ratio of non-HDL-c to HDL-c has been identified as a risk factor for the development of cardiovascular disease across various populations [62]. Çelik et al. determined that the levels of AC were considerably elevated in comparison to the control group, with the exception of the HDL-c value, which was notably reduced relative to the control group. This underscores the role of the atherosclerotic component, recognized as the predominant cause of cardiovascular diseases, which represents a complex, inflammatory, and fibroproliferative response induced by the accumulation of atherogenic lipoproteins within the arterial intima [63].

Adedokun et al. demonstrated a significant difference in AC among the subgroups with abnormal lipid profiles [64]. Our research shows notable differences between the AC subgroups, including both PreDM and T2DM patients, with respect to TC, LDL-c, and HDL-c levels.

In the study conducted by Wan et al., they identified an increase in waist, increased WC, WHR, and the Chinese visceral adiposity index (CVAI), all of which were significantly associated with a higher prevalence of DKD in both males and females [65], while our study revealed elevated values of VAI when comparing the PreDM group with T2DM group. Simultaneously, we achieved statistical significance for VAI in subgroups where VAI values were notably higher. Specifically, the subgroup with an AC ≥ 3.05 exhibited a statistical *p*-value of 0.002, while the subgroup with an AC ≥ 2.74 had a *p*-value of 0.036. Furthermore, in the diabetic subgroup with a PNI value below 48.94, the median VAI was significantly higher (*p* = 0.08).

An observational study carried out within an Asian population revealed that the prevalence of DKD among individuals diagnosed with type 2 diabetes is as high as 58.6%. This condition is acknowledged as a significant contributor to chronic kidney disease and end-stage renal disease [66]. Additionally, our study revealed a statistical difference in eGFR, as calculated using the CKD-EPI equation. Initially, a significant difference in eGFR between female and male patient clusters was observed in both PreDM and T2DM groups (*p* = 0.039 and *p* < 0.0001, respectively). Moreover, it was noted that an increase in the atherogenic coefficient corresponded to a decrease in eGFR among T2DM patients. Furthermore, it is noteworthy that the lowest mean eGFR recorded was 43.92 mL/min/1.73 m^2^ when comparing the prediabetic patient group to the group of individuals diagnosed with T2DM.

VAI was demonstrated to be an independent predictor of T2DM; He et. all also stated that screening may enhance early detection and prevention strategies for diabetes [67]. Over the past three decades, epidemiological studies have shown that visceral adipose tissue (VAT), particularly ectopic fat accumulation, is independently linked to an increased risk of T2DM. Research has also demonstrated that visceral adiposity serves as a marker of atherosclerotic burden in dysmetabolic patients, while subcutaneous fat is thought to offer protection against atherosclerotic plaques. A higher VAI has been associated with greater atherosclerotic burden. While imaging methods like CT, MRI, and DEXA are considered gold standards for quantifying VAT and subcutaneous adipose tissue (SAT), their high cost and inconvenience make them unsuitable for large-scale studies. As a result, non-imaging clinical methods such as waist circumference, waist-to-height ratio, and waist-to-hip ratio have been developed, but these anthropometric indices cannot effectively differentiate between VAT and SAT on their own [68,69].

Bullen et al. concluded in their study that an increase in the value of the VAI is correlated with a progressive decline in eGFR. Furthermore, the researchers emphasized the correlation between obesity metrics and the occurrence of kidney failure, observing that the occurrence increased with rising quartiles of VAI [70]. Another study highlighted the VAI as a significant risk factor for the development of DKD, with elevated VAI levels strongly associated with its onset in a representative cohort of US adults with diabetes. Furthermore, VAI may serve as a superior indicator, unlike BMI, for predicting the development of DKD [71]. A significant difference was also observed in our study, regarding these parameters in the comparison between the PreDM and T2DM cohorts.

The findings of our study indicate the presence of sex-specific differences and trends, where a correlation was observed between elevated values of VAI and reduced values of eGFR. This discovery is supported by research conducted by Kim et al., which demonstrates a significant decreasing trend in eGFR alongside an opposing trend in VAI values [5].

A Chinese study indicated that among females, all anthropometric indices, excluding BMI, were significantly larger in subjects diagnosed with CKD compared to those without CKD. Furthermore, notable differences were observed in TC, TG, LDL-c, and FPG [72]. The current study has revealed elevated means or medians for BAI, VAI, and LDL-c.

Although our study did not employ the research of molecular markers, it is of utmost importance to mention Li et al.’s findings: the interplay between chronic inflammation and endothelial dysfunction in diabetic nephropathy is critical in the pathogenesis of atherosclerosis. Elevated inflammatory mediators such as interleukin 1 β (IL-1β) and tumor necrosis factor α (TNF-α) activate the NF-κB signaling pathway, leading to the upregulation of adhesion molecules and pro-inflammatory cytokines. This cascade promotes the accumulation of foam cells and the formation of atherosclerotic plaques, thereby worsening renal and cardiovascular complications [73].

The underlying mechanisms linking VAI and FPG may involve several key factors. Specifically, an excess of visceral fat can lead to the heightened secretion of pro-inflammatory adipokines, such as interleukin-6 (IL-6) and leptin. This dysregulation may play a significant role in the development of insulin resistance and the pathogenesis of diabetes [74]. Nasser et al. identified VAI as the sole determinant of adiponectin levels, which is recognized as the primary protective adipokine exhibiting anti-diabetogenic properties [75].

This study, conducted at a single university clinical hospital in Dolj County, reveals significant limitations tied to its design. The absence of a pilot study and pertinent baseline data from the existing literature hinder the precise estimation of effect size, ultimately precluding any sample size simulation. The relatively small and geographically localized sample may not fully represent the broader diabetic population, limiting the generalizability of the findings.

The rarity of patients with severe complications, reduced willingness to pursue medical care, or mobility impairments is anticipated, which may introduce selection bias into our findings. Furthermore, the limited sample size among specific subgroups post-classification could notably influence the study outcomes.

The study was not designed to explore the underlying molecular mechanisms linking atherogenic dyslipidemia to renal damage, warranting further experimental studies. We are looking forward to investigating more in our future studies, researching markers such as proprotein convertase subtilisin/kexin type 9, soluble epoxide hydrolase 2, advanced oxidation protein product and thiobarbituric acid reactive substances.

Therefore, further research is warranted to investigate the associations between obesity-related indices, the AC, and the PNI across diverse patient categories with diabetes and prediabetes.

## 5. Conclusions

The study revealed significant differences between patients with T2DM and those with PreDM in terms of demographic, clinical, metabolic, and nutritional characteristics. Demographically, T2DM patients were significantly older, with a mean age of 64.25 years compared to 48.60 years in the PreDM group (*p* < 0.0001). However, no significant differences were found in gender distribution or place of residence between the two groups.

Metabolic and laboratory markers indicated more severe dysregulation in T2DM patients. Key diabetes-related biomarkers such as FPG, 2hPG, and HbA1c were significantly elevated in the T2DM group compared to PreDM (*p* < 0.0001). Additionally, kidney function deterioration was evident, as eGFR was significantly lower in the T2DM group (*p* < 0.0001).

Obesity and cardiovascular risk factors were also more pronounced in T2DM patients. There was a significantly higher mean weight (*p* = 0.047) and BMI in the patients with obesity category (*p* = 0.01), along with an elevated VAI (*p* = 0.04). Furthermore, AC, a marker of cardiovascular risk, was significantly higher in the T2DM group (*p* = 0.001), indicating a greater likelihood of cardiovascular complications, hence microvascular complications that may aggravate DN.

Lastly, nutritional and inflammatory markers showed that T2DM patients experienced higher systemic inflammation and a decline in nutritional status. The levels of CRP were significantly elevated in the T2DM group (*p* < 0.0001), signaling increased inflammation. Meanwhile, the PNI was significantly lower in these patients (*p* < 0.0001), suggesting a higher prevalence of mild malnutrition. These findings highlight the complex interplay between metabolic dysfunction, obesity, cardiovascular risk, and inflammation in diabetes progression.

## Figures and Tables

**Table 1:** Clinical and demographic characteristics of patients diagnosed with prediabetes and diabetes.

Features	PreDM Cohort (*n* = 50)	T2DM Cohort (*n* = 48)	*p*-Value from Pearson’s Chi-Squared/ Student’s *t*-Test
*Demographic features*			
Age (years) (mean ± SD)	48.60 ± 7.68	64.25 ± 11.95	<0.0001 *
Gender, female/male (*n*)	30/20	24/24	0.319
Residence, rural/urban (*n*)	16/34	21/27	0.230
*Medical history and clinical condition*			
Smoking history, no/yes (*n*)	18/32	23/25	0.231
Drinking history, no/yes (*n*)	14/36	20/28	0.155
Education, no/yes (*n*)	12/38	19/29	0.097
Hypertension, *n* (%)	40 (80%)	44 (91%)	0.098
Dyslipidemia, *n* (%)	43 (86%)	41 (85%)	0.934
Hepatosteatosis, *n* (%)	35 (70%)	33 (68%)	0.893
SBP (mmHg) (mean ± SD)	132.60 ± 16.10	136.1 ± 19.48	0.327
DBP (mmHg) (mean ± SD)	79.04 ± 13.30	79.36 ± 14.02	0.908
Height (cm) (mean ± SD)	167 ± 11	167 ± 10	0.761
Weight (kg) (mean ± SD)	82.40 ± 20.56	87.68 ± 17.34	0.047 *
WC (cm) (mean ± SD)	101.9 ± 17.51	105.80 ± 12.73	0.204
HC (cm) (mean ± SD)	107.80 ± 13.10	110.40 ± 15.09	0.365
WHR [median (range)]	0.94 (0.59–1.64)	0.94 (0.78–3.33)	0.623
WHtR (mean ± SD)	0.60 ± 0.09	0.63 ± 0.08	0.179
BMI (kg/m^2^) (mean ± SD)	30.42 ± 6.50	31.18 ± 5.13	0.518
BMI category (*n*)			
Normal (18.5–24.9 kg/m^2^)	11	6	0.443
Overweight (25–29.9 kg/m^2^)	20	12	0.735
Obese (≥30 kg/m^2^)	19	30	0.01 *
BAI (mean ± SD)	32.17 ± 6.87	33.23 ± 8.56	0.501
VAI [median (range)]	3.74 (1.05–35.13)	4.85 (1.21–28.59)	0.04 *
*Laboratory examination*			
FPG (mg/dL) (mean ± SD)	107.30 ± 5.68	159 ± 24.43	<0.0001 *
2hPG (mg/dL) (mean ± SD)	169.10 ± 15.12	245.1 ± 60.72	<0.0001 *
HbA1c (%) (mean ± SD)	5.79 ± 0.47	9.80 ± 1.80	<0.0001 *
TC (mg/dL) (mean ± SD)	182.90 ± 48.92	192.4 ± 60.99	0.395
TG (mg/dL) (mean ± SD)	125.70 ± 73.08	151.5 ± 93.14	0.130
LDL-c (mg/dL) (mean ± SD)	107.90 ± 43.97	105.8 ± 38.45	0.797
HDL-c (mg/dL) (mean ± SD)	52.18 ± 13.84	49.38 ± 12.19	0.290
eGFR (mL/min/1.73 m^2^)			
CKD-EPI (mL/min/1.73 m^2^) (mean ± SD)	90.18 ± 26.70	43.92 ± 12.67	<0.0001 *
BUN (mg/dL) (mean ± SD)	50.63 ± 14.98	27.71 ± 13.45	<0.0001 *
Creatinine (mg/dL) (mean ± SD)	0.91 ± 0.38	1.59 ± 0.27	<0.0001 *
UA (mg/dL) (mean ± SD)	4.91 ± 1.51	4.84 ± 1.77	0.831
Hb (g/dL) (mean ± SD)	13.61 ± 2.05	13.73 ± 2.13	0.778
WBC (×10^3^/μL) (mean ± SD)	7.57 ± 2.04	7.72 ± 1.86	0.695
NEU (×10^3^/μL) (mean ± SD)	4.69 ± 1.55	4.83 ± 1.52	0.672
LYM (×10^3^/μL) (mean ± SD)	2.14 ± 0.71	2.13 ± 0.74	0.950
MON (×10^3^/μL) [median (range)]	0.46 (0.25–0.95)	0.49 (0.32–1.19)	0.631
PLT (×10^3^/μL) (mean ± SD)	256.3 ± 78.63	257.9 ± 69.17	0.911
*Malnourishment*			
ALB (g/dL) (mean ± SD)	6.19 ± 0.51	3.82 ± 0.27	<0.0001 *
CRP (mg/dL) [median (range)]	0.49 (0.05–162.1)	20.70 (3.20–76)	<0.0001 *
ESR (mm/1st h) [median (range)]	30 (8–115)	29 (4–110)	0.902
PNI (mean ± SD)	72.69 ± 6.81	48.94 ± 5.25	<0.0001 *
AC (mean ± SD)	2.74 ± 1.46	3.05 ± 1.52	0.001 *

2hPG: two-hour plasma glucose after a 75 g oral glucose tolerance test; AC: atherogenic coefficient; ALB: albumin; BAI: body adiposity index; BMI: body mass index; BUN: blood urea nitrogen; CKD-EPI: chronic kidney disease epidemiology collaboration; CREA: creatinine; CRP: C-reactive protein; DBP: diastolic blood pressure; e-GFR: estimated glomerular filtration rate; ESR: erythrocyte sedimentation rate; FPG: fasting plasma glucose; Hb: hemoglobin; HbA1c: glycosylated hemoglobin A1c; HC: hip circumference; HDL-c: high-density lipoprotein cholesterol; LDL-c: low-density lipoprotein cholesterol; LYM: lymphocytes; MON: monocytes; NEU: neutrophils; PLT: platelets; PNI: prognostic nutritional index; SBP: systolic blood pressure; SD: standard deviation; TC: total cholesterol; TG: total triglycerides; UA: uric acid; WBC: white blood cells/leukocytes; WC: waist circumference; WHR: waist-to-hip ratio; WHtR: waist-to-height ratio. * *p* < 0.05: statistically significant.

**Table 2:** Comparative analysis of the clinical features between the AC and PNI groups in the context of prediabetes.

Features		PreDM Cohort (*n* = 50)					
		**PNI**			**AC**		
	All Patients	PNI ≥ 72.69	PNI < 72.69	*p*-Value	*AC* < 2.74	*AC* ≥ 2.74	*p*-Value
Patients (*n*)	50	26	24		31	19	
*Demographic features*							
Age (years) (mean ± SD)	48.60 ± 7.68	48.31 ± 8.38	48.92 ± 7.02	0.782	48.84 ± 7.79	48.21 ± 7.70	0.782
Gender, female/male (*n*)	30/20	14/12	16/8	0.355	21/10	9/10	0.153
Residence, rural/urban (*n*)	16/34	11/15	9/15	0.728	7/24	9/10	0.068
*Medical history and clinical condition*							
Smoking history, no/yes (*n*)	18/32	10/16	8/16	0.705	12/19	6/13	0.610
Drinking history, no/yes (*n*)	14/36	12/14	2/22	0.002 *	13/18	1/18	0.005 *
Education, no/yes (*n*)	12/38	10/16	8/16	0.705	5/26	7/12	0.095
Hypertension, *n* (%)	40 (80%)	18 (69%)	22 (91%)	0.047 *	22 (71%)	18 (94%)	0.041 *
Dyslipidemia, *n* (%)	43 (86%)	20 (77%)	23 (95%)	0.054 **	26 (83%)	17 (89%)	0.579
Hepatosteatosis, *n* (%)	35 (70%)	17 (65%)	16 (66%)	0.923	21 (67%)	14 (73%)	0.656
SBP (mmHg) (mean ± SD)	132.60 ± 16.10	130.2 ± 13.17	135.2 ± 18.72	0.275	133.9 ± 16.63	130.5 ± 15.40	0.470
DBP (mmHg) (mean ± SD)	79.36 ± 14.02	76.85 ± 11.74	82.08 ± 15.95	0.190	78.68 ± 13.91	80.47 ± 14.53	0.664
Height (cm) (mean ± SD)	167 ± 11	168 ± 10	165 ± 11	0.423	166 ± 10	168 ± 11	0.486
Weight (kg) (mean ± SD)	82.40 ± 20.56	76.32 ± 13.96	90.67 ± 21.70	0.007 *	81.67 ± 21.65	85.72 ± 14.94	0.477
WC (cm) (mean ± SD)	101.9 ± 17.51	101.3 ± 13.93	102.3 ± 20.54	0.840	98.39 ± 15.06	107.5 ± 20.05	0.042 *
HC (cm) (mean ± SD)	107.80 ± 13.10	105.2 ± 11.56	110.5 ± 14.32	0.048 *	107.4 ± 14.71	108.3 ± 10.30	0.817
WHR [median (range)]	0.94 (0.59–1.64)	0.92 (0.78–1.17)	0.96 (0.59–1.64)	0.064	0.92 (0.59–1.17)	0.97 (0.84–1.64)	0.115
WHtR (mean ± SD)	0.60 ± 0.09	0.60 ± 0.10	0.61 ± 0.07	0.870	0.59 ± 0.08	0.63 ± 0.09	0.056 **
BMI (kg/m^2^) (mean ± SD)	30.42 ± 6.50	28.26 ± 5.97	32.75 ± 6.35	0.013 *	30.57 ± 7.17	30.16 ± 5.38	0.829
BMI category (*n*)							
Normal (18.5–24.9 kg/m^2^)	11	8	3	0.026 *	7	4	0.930
Overweight (25–29.9 kg/m^2^)	20	13	7	0.306	13	7	0.525
Obese (≥30 kg/m^2^)	19	5	14	0.473	11	8	0.153
BAI (mean ± SD)	32.17 ± 6.87	30.48 ± 6.22	34.01 ± 7.20	0.068	32.38 ± 7.47	31.84 ± 5.96	0.788
VAI [median (range)]	3.74 (1.05–35.13)	3.74 (1.05–35.13)	3.80 (1.24–9.74)	0.890	3.54 (1.05–9.29)	3.94 (1.24–35.13)	0.036 *
*Laboratory examination*							
FPG (mg/dL) (mean ± SD)	107.30 ± 5.68	106.8 ± 5.44	107.8 ± 6.02	0.579	107.2 ± 5.91	107.4 ± 5.46	0.892
2hPG (mg/dL) (mean ± SD)	169.10 ± 15.12	168.5 ± 15.15	169.7 ± 15.39	0.795	171 ± 14.98	165.9 ± 15.22	0.255
HbA1c (%) (mean ± SD)	5.79 ± 0.47	5.74 ± 0.52	5.84 ± 0.42	0.453	5.85 ± 0.47	5.70 ± 0.46	0.309
TC (mg/dL) (mean ± SD)	182.90 ± 48.92	183.1 ± 52.09	182.7 ± 46.35	0.979	167.1 ± 44.62	208.6 ± 45.48	0.002 *
TG (mg/dL) (mean ± SD)	125.70 ± 73.08	120.5 ± 88.36	131.4 ± 53.18	0.602	120.3 ± 53.95	134.6 ± 97.75	0.506
LDL-c (mg/dL) (mean ± SD)	107.90 ± 43.97	107.5 ± 41.68	108.3 ± 46.80	0.954	93.30 ± 38.31	131.7 ± 43.02	0.001 *
HDL-c (mg/dL) (mean ± SD)	52.18 ± 13.84	55.52 ± 12.68	49.10 ± 14.37	0.048 *	58.79 ± 12.83	41.40 ± 6.98	<0.0001 *
CKD-EPI (mL/min/1.73 m^2^) (mean ± SD)	90.18 ± 26.70	90.92 ± 19.40	89.51 ± 32.41	0.854	89.99 ± 27.78	90.51 ± 25.59	0.947
BUN (mg/dL) (mean ± SD)	50.63 ± 14.98	50.10 ± 16.49	51.20 ± 13.48	0.798	53.38 ± 13.58	46.14 ± 16.39	0.057 **
Creatinine (mg/dL) (mean ± SD)	0.91 ± 0.38	0.86 ± 0.25	0.96 ± 0.48	0.382	0.92 ± 0.40	0.91 ± 0.36	0.971
UA (mg/dL) (mean ± SD)	4.91 ± 1.51	4.81 ± 1.59	5.03 ± 1.44	0.609	4.76 ± 1.30	5.16 ± 1.80	0.373
Hb (g/dL) (mean ± SD)	13.61 ± 2.05	14.54 ± 1.34	12.76 ± 2.24	0.001 *	13.56 ± 1.61	13.69 ± 2.68	0.830
WBC (×10^3^/μL) (mean ± SD)	7.57 ± 2.04	6.71 ± 1.66	8.49 ± 2.03	0.001 *	8.04 ± 2.05	6.79 ± 1.81	0.033 *
NEU (×10^3^/μL) (mean ± SD)	4.69 ± 1.55	4.23 ± 1.25	5.20 ± 1.70	0.025 *	5.08 ± 1.64	4.07 ± 1.16	0.023 *
LYM (×10^3^/μL) (mean ± SD)	2.14 ± 0.71	1.81 ± 0.46	2.50 ± 0.77	0.0004 *	2.19 ± 0.70	2.05 ± 0.73	<0.0001 *
MON (×10^3^/μL) [median (range)]	0.46 (0.25–0.95)	0.43 (0.25–0.95)	0.52 (0.25–0.82)	0.025 *	0.50 (0.25–0.83)	0.42 (0.25–0.95)	0.154
PLT (×10^3^/μL) (mean ± SD)	256.3 ± 78.63	261 ± 83.81	251.1 ± 74.06	0.660	256.7 ± 76.12	255.5 ± 84.70	0.958
*Malnourishment*							
ALB (g/dL) (mean ± SD)	6.19 ± 0.51	6.55 ± 0.43	5.86 ± 0.31	<0.0001 *	6.18 ± 0.39	6.22 ± 0.66	0.762
CRP (mg/dL) [median (range)]	20.70 (3.20–76)	20.45 (6–76)	21.50 (3.2–54)	0.606	26 (6–58)	17 (3.2–76)	0.052 **
ESR (mm/1st h) [median (range)]	30 (8–115)	29.50 (8–96)	35 (10–115)	0.250	30 (10–115)	30 (8–105)	0.830
PNI (mean ± SD)	72.69 ± 6.81	78.03 ± 5.23	67.76 ± 3.64	<0.0001 *	72.78 ± 4.93	72.55 ± 9.27	0.908
AC (mean ± SD)	2.74 ± 1.46	2.41 ± 1.04	3.04 ± 1.73	0.055 **	1.88 ± 0.66	4.13 ± 1.35	<0.0001 *

2hPG: two-hour plasma glucose after a 75 g oral glucose tolerance test; AC: atherogenic coefficient; ALB: albumin; BAI: body adiposity index; BMI: body mass index; BUN: blood urea nitrogen; CKD-EPI: chronic kidney disease epidemiology collaboration; CREA: creatinine; CRP: C-reactive protein; DBP: diastolic blood pressure; e-GFR: estimated glomerular filtration rate; ESR: erythrocyte sedimentation rate; FPG: fasting plasma glucose; Hb: hemoglobin; HbA1c: glycosylated hemoglobin A1c; HC: hip circumference; HDL-c: high-density lipoprotein cholesterol; LDL-c: low-density lipoprotein cholesterol; LYM: lymphocytes; MON: monocytes; NEU: neutrophils; PLT: platelets; PNI: prognostic nutritional index; SBP: systolic blood pressure; SD: standard deviation; TC: total cholesterol; TG: total triglycerides; UA: uric acid; WBC: white blood cells/leukocytes; WC: waist circumference; WHR: waist-to-hip ratio; WHtR: waist-to-height ratio. * *p* < 0.05: statistically significant; **: stretched the significance limit.

**Table 3:** Comparative analysis of the clinical features between the AC and PNI groups in the context of diabetes.

Features	T2DM Cohort (*n* = 48)						
		PNI			AC		
	All Patients	PNI ≥ 48.94	PNI < 48.94	*p*-Value	*AC* < 3.05	*AC* ≥ 3.05	*p*-Value
Patients (*n*)	48	23	25		29	19	
*Demographic features*							
Age (years) (mean ± SD)	64.25 ± 11.95	67.43 ± 11.28	61.32 ± 12.02	0.076	66.62 ± 11.08	60.63 ± 12.62	0.089
Gender, female/male (*n*)	24/24	11/12	13/12	0.772	18/11	6/13	0.038 *
Residence, rural/urban (*n*)	21/27	13/10	8/17	0.087	14/15	7/12	0.434
*Medical history and clinical condition*							
Smoking history, no/yes (*n*)	23/25	9/14	7/18	0.413	13/16	6/13	0.358
Drinking history, no/yes (*n*)	20/28	14/9	10/15	0.148	12/17	8/11	0.960
Education, no/yes (*n*)	19/29	10/13	11/14	0.970	12/17	5/14	0.285
Hypertension, *n* (%)	44 (91%)	22 (95%)	22 (88%)	0.337	26 (89%)	18 (94%)	0.533
Dyslipidemia, *n* (%)	41 (85%)	19 (82%)	22 (88%)	0.597	24 (82%)	17 (89%)	0.519
Hepatosteatosis, *n* (%)	33 (68%)	16 (69%)	17 (68%)	0.906	17 (58%)	16 (84%)	0.061
SBP (mmHg) (mean ± SD)	136.1 ± 19.48	134.2 ± 19.32	138 ± 19.84	0.507	132.6 ± 20.02	141.6 ± 17.74	0.115
DBP (mmHg) (mean ± SD)	79.04 ± 13.30	77.09 ± 14.14	80.84 ± 12.50	0.334	76.31 ± 14.44	83.21 ± 10.37	0.078
Height (cm) (mean ± SD)	167 ± 10	168 ± 8	167 ± 11	0.636	165 ± 8	170 ± 11	0.104
Weight (kg) (mean ± SD)	87.68 ± 17.34	85.71 ± 16.58	89.48 ± 18.15	0.045	85.22 ± 13.30	91.43 ± 22.02	0.229
WC (cm) (mean ± SD)	105.80 ± 12.73	104.3 ± 13.33	107.5 ± 12.12	0.381	107.9 ± 12.87	102.7 ± 12.18	0.169
HC (cm) (mean ± SD)	110.40 ± 15.09	108.8 ± 18.42	112 ± 10.50	0.046 *	112.8 ± 11.03	106.7 ± 19.53	0.174
WHR [median (range)]	0.94 (0.78–3.33)	0.92 (0.78–1.11)	0.96 (0.85–3.33)	0.133	0.92 (0.80–1.11)	0.96 (0.78–3.33)	0.047 *
WHtR (mean ± SD)	0.63 ± 0.08	0.62 ± 8.55	0.63 ± 0.07	0.581	0.60 ± 0.07	0.65 ± 0.07	0.040 *
BMI (kg/m^2^) (mean ± SD)	31.18 ± 5.13	30.27 ± 4.42	32.03 ± 5.66	0.238	31.13 ± 4.71	31.22 ± 5.85	0.949
BMI category (*n*)							
Normal (18.5–24.9 kg/m^2^)	6	3	3	0.070	3	3	0.468
Overweight (25–29.9 kg/m^2^)	12	7	5	0.017 *	7	5	0.866
Obese (≥30 kg/m^2^)	30	13	17	0.047 *	19	11	0.399
BAI (mean ± SD)	33.23 ± 8.56	33.06 ± 10.82	33.42 ± 5.38	0.884	30.49 ± 11.08	35.03 ± 5.99	0.039 *
VAI [median(range)]	4.85 (1.21–28.59)	3.98 (1.21–10.31)	6.31 (1.38–28.59)	0.008 *	4.31 (1.21–7.83)	7.62 (2.43–28.59)	0.002 *
*Laboratory examination*							
FPG (mg/dL) (mean ± SD)	159 ± 24.43	154.5 ± 20.72	163.9 ± 27.55	0.188	157 ± 29.11	162.1 ± 14.94	0.481
2hPG (mg/dL) (mean ± SD)	245.1 ± 60.72	228.7 ± 45.71	262.9 ± 70.44	0.049 *	244.7 ± 69.88	245.7 ± 45.06	0.956
HbA1c (%) (mean ± SD)	9.80 ± 1.80	9.48 ± 1.66	10.13 ± 1.92	0.021 *	9.68 ± 1.97	9.96 ± 1.54	0.048 *
TC (mg/dL) (mean ± SD)	192.4 ± 60.99	169.1 ± 52.18	213.9 ± 61.54	0.009 *	161.9 ± 42.29	239 ± 56.04	<0.0001 *
TG (mg/dL) (mean ± SD)	151.5 ± 93.14	111.4 ± 49.40	188.3 ± 108.6	0.003 *	119.6 ± 52.18	200.1 ± 119.5	0.002 *
LDL-c (mg/dL) (mean ± SD)	105.8 ± 38.45	95.42 ± 34.55	115.3 ± 40.05	0.035 *	85.78 ± 28.13	136.3 ± 31.70	<0.0001 *
HDL-c (mg/dL) (mean ± SD)	49.38 ± 12.19	47.31 ± 11.30	51.28 ± 12.89	0.064	52.52 ± 12.87	44.59 ± 9.49	0.025 *
CKD-EPI (mL/min/1.73 m^2^) (mean ± SD)	43.92 ± 12.67	44.56 ± 12.42	43.23 ± 13.19	0.720	45.87 ± 11.68	40.65 ± 13.33	0.039 *
BUN (mg/dL) (mean ± SD)	27.71 ± 13.45	27.42 ± 12.57	28.01 ± 14.61	0.880	29.09 ± 13.31	25.59 ± 13.74	0.382
Creatinine (mg/dL) (mean ± SD)	1.59 ± 0.27	1.58 ± 0.23	1.61 ± 0.32	0.702	1.58 ± 0.30	1.62 ± 0.24	0.651
UA (mg/dL) (mean ± SD)	4.84 ± 1.77	4.83 ± 2.02	4.86 ± 1.54	0.951	4.38 ± 1.87	5.55 ± 1.34	0.023 *
Hb (g/dL) (mean ± SD)	13.73 ± 2.13	14.46 ± 1.58	12.94 ± 2.38	0.011 *	13.26 ± 1.50	14.45 ± 2.72	0.057 **
WBC (×10^3^/μL) (mean ± SD)	7.72 ± 1.86	8.30 ± 1.67	7.09 ± 1.89	0.023 *	7.26 ± 1.67	8.42 ± 1.97	0.035 *
NEU (×10^3^/μL) (mean ± SD)	4.83 ± 1.52	4.86 ± 1.37	4.79 ± 1.70	0.878	4.52 ± 1.14	5.30 ± 1.91	0.047 *
LYM (×10^3^/μL) (mean ± SD)	2.13 ± 0.74	2.61 ± 0.63	1.60 ± 0.43	<0.0001 *	2.03 ± 0.77	2.27 ± 0.69	0.279
MON (×10^3^/μL) [median (range)]	0.49 (0.32–1.19)	0.48 (0.32–1.19)	0.50 (0.34–1.06)	0.598	0.50 (0.32–0.68)	0.49 (0.35–1.19)	0.142
PLT (×10^3^/μL) (mean ± SD)	257.9 ± 69.17	272.8 ± 50.25	241.8 ± 83.33	0.122	252.1 ± 71.58	266.9 ± 66.20	0.473
*Malnourishment*							
ALB (g/dL) (mean ± SD)	3.82 ± 0.27	3.95 ± 0.28	3.68 ± 0.19	0.0004 *	3.84 ± 0.29	3.80 ± 0.25	0.691
CRP (mg/dL) [median (range)]	0.49 (0.05–162.1)	0.48 (0.09–162.1)	0.51 (0.05–35)	0.439	0.31 (0.05–36)	0.81 (0.09–162.1)	0.023 *
ESR (mm/1st h) [median (range)]	29 (4–110)	29 (4–105)	29 (5–110)	0.433	25 (4–110)	35 (5–105)	0.059 **
PNI (mean ± SD)	48.94 ± 5.25	52.64 ± 4.16	44.91 ± 2.72	<0.0001 *	48.59 ± 5.50	49.47 ± 4.94	0.578
AC (mean ± SD)	3.05 ± 1.52	2.63 ± 0.96	3.44 ± 1.84	0.064	2.10 ± 0.47	4.50 ± 1.43	<0.0001 *

2hPG: two-hour plasma glucose after a 75 g oral glucose tolerance test; AC: atherogenic coefficient; ALB: albumin; BAI: body adiposity index; BMI: body mass index; BUN: blood urea nitrogen; CKD-EPI: chronic kidney disease epidemiology collaboration; CREA: creatinine; CRP: C-reactive protein; DBP: diastolic blood pressure; e-GFR: estimated glomerular filtration rate; ESR: erythrocyte sedimentation rate; FPG: fasting plasma glucose; Hb: hemoglobin; HbA1c: glycosylated hemoglobin A1c; HC: hip circumference; HDL-c: high-density lipoprotein cholesterol; LDL-c: low-density lipoprotein cholesterol; LYM: lymphocytes; MON: monocytes; NEU: neutrophils; PLT: platelets; PNI: prognostic nutritional index; SBP: systolic blood pressure; SD: standard deviation; TC: total cholesterol; TG: total triglycerides; UA: uric acid; WBC: white blood cells/leukocytes; WC: waist circumference; WHR: waist-to-hip ratio; WHtR: waist-to-height ratio. * *p* < 0.05: statistically significant; **: stretched the significance limit.

**Table 4:** Examination of the associations between AC and *BAI*, *VAI*, *WHR*, *WHtR*, and *BMI* within the prediabetes (PreDM) and type 2 diabetes mellitus (T2DM) populations.

	Variables (Mean ± SD)	PreDM Group (*n* = 50)		T2DM Group (*n* = 48)	
		AC	*p*-Value from Kruskal–Wallis/ One-Way ANOVA	AC	*p*-Value from Kruskal–Wallis/ One-Way ANOVA
	BMI category (kg/m^2^)				
	Normal weight (18.5–24.9 kg/m^2^)	2.53 ± 1.99	0.874	3.30 ± 1.86	0.826
	Overweight (25–29.9 kg/m^2^)	2.78 ± 0.92		2.84 ± 1.19	
	Obese (≥30 kg/m^2^)	2.81 ± 1.64		3.08 ± 1.61	
	WHR				
	Q 1	2.82 ± 1.85	0.039 *	2.31 ± 0.68	0.042 *
PreDM Value	(0.59–0.86)				
T2DM Value	(0.78–0.89)				
	Q 2	2.21 ± 0.73		2.79 ± 1.30	
PreDM Value	(0.87–0.93)				
T2DM Value	(0.90–0.93)				
	Q 3	2.72 ± 1.46		3.11 ± 1.07	
PreDM Value	(0.94–0.97)				
T2DM Value	(0.94–0.99)				
	Q 4	3.13 ± 1.58		4.11 ± 2.26	
PreDM Value	(0.98–1.64)				
T2DM Value	(1.00–3.33)				
	WHtR				
	Q 1	2.46 ± 0.93	0.054 **	2.22 ± 0.83	0.049 *
PreDM Value	(0.33–0.55)				
T2DM Value	(0.41–0.57)				
	Q 2	2.44 ± 1.22		3.05 ± 0.99	
PreDM Value	(0.56–0.59)				
T2DM Value	(0.58–0.61)				
	Q 3	2.83 ± 1.18		3.14 ± 1.24	
PreDM Value	(0.60–0.64)				
T2DM Value	(0.62–0.67)				
	Q 4	3.20 ± 2.26		3.78 ± 2.30	
PreDM Value	(0.65–0.95)				
T2DM Value	(0.68–0.79)				
	BAI				
	Q 1	2.81 ± 0.93	0.619	2.55 ± 0.91	0.033 *
PreDM Value	(15.84–26.84)				
T2DM Value	(2.84–28.93)				
	Q 2	2.43 ± 1.20		2.58 ± 1.11	
PreDM Value	(26.85–30.64)				
T2DM Value	(28.94–32.70)				
	Q 3	3.15 ± 1.77		2.97 ± 0.84	
PreDM Value	(30.65–37.00)				
T2DM Value	(32.71–39.73)				
	Q 4	2.55 ± 1.83		4.10 ± 2.33	
PreDM Value	(37.01–48.93)				
T2DM Value	(39.74–52.86)				
	VAI				
	Q 1	2.09 ± 0.81	0.037 *	2.09 ± 0.94	<0.0001 *
PreDM Value	(1.05–2.73)				
T2DM Value	(1.21–3.27)				
	Q 2	2.83 ± 2.16		2.74 ± 0.67	
PreDM Value	(2.74–3.73)				
T2DM Value	(3.28–4.84)				
	Q 3	2.95 ± 1.21		2.62 ± 0.75	
PreDM Value	(3.74–5.46)				
T2DM Value	(4.85–7.57)				
	Q 4	3.04 ± 1.25		4.75 ± 1.88	
PreDM Value	(5.47–35.13)				
T2DM Value	(7.58–28.59)				

Q1: quarter 1; Q2: quarter 2; Q3: quarter 3: Q4: quarter 4; BAI: body adiposity index; VAI: visceral adiposity index; WHR: waist-to-hip ratio; WHtR: waist-to-height ratio; BMI: body mass index; * *p* < 0.05: statistically significant; **: stretched the significance limit.

**Table 5:** Examination of the associations between PNI and *BAI*, *VAI*, *WHR*, *WHtR*, and *BMI* within the prediabetes (PreDM) and type 2 diabetes mellitus (T2DM) populations.

	Variables (Mean ± SD)	PreDM Group (*n* = 50)		T2DM Group (*n* = 48)	
		PNI	*p*-Value from Kruskal–Wallis/ One-Way ANOVA	PNI	*p*-Value from Kruskal–Wallis/ One-Way ANOVA
	BMI category (kg/m^2^)				
	Normal weight (18.5–24.9 kg/m^2^)	69.92 ± 7.07	0.002 *	48.27 ± 5.09	0.860
	Overweight (25–29.9 kg/m^2^)	70.37 ± 4.47		47.35 ± 4.03	
	Obese (≥30 kg/m^2^)	76.74 ± 7.03		48.19 ± 6.87	
	WHR				
	Q 1	73.15 ± 5.16	0.555	51.02 ± 5.99	0.355
PreDM Value	(0.59–0.86)				
T2DM Value	(0.78–0.89)				
	Q 2	75.03 ± 8.18		49.03 ± 4.08	
PreDM Value	(0.87–0.93)				
T2DM Value	(0.90–0.93)				
	Q 3	72.04 ± 5.17		47.12 ± 5.49	
PreDM Value	(0.94–0.97)				
T2DM Value	(0.94–0.99)				
	Q 4	70.85 ± 8		48.77 ± 5.26	
PreDM Value	(0.98–1.64)				
T2DM Value	(1.00–3.33)				
	WHtR				
	Q 1	71.89 ± 4.67	0.221	49.29 ± 5.2	0.419
PreDM Value	(0.33–0.55)				
T2DM Value	(0.41–0.57)				
	Q 2	72.17 ± 5.86		50.45 ± 6.77	
PreDM Value	(0.56–0.59)				
T2DM Value	(0.58–0.61)				
	Q 3	73.92 ± 8.4		46.95 ± 5.29	
PreDM Value	(0.60–0.64)				
T2DM Value	(0.62–0.67)				
	Q 4	72.58 ± 8.02		49.35 ± 3.48	
PreDM Value	(0.65–0.95)				
T2DM Value	(0.68–0.79)				
	BAI				
	Q 1	69.87 ± 6.20	0.296	49.12 ± 5.23	0.034 *
PreDM Value	(15.84–26.84)				
T2DM Value	(2.84–28.93)				
	Q 2	72.97 ± 5.35		48.36 ± 6.23	
PreDM Value	(26.85–30.64)				
T2DM Value	(28.94–32.70)				
	Q 3	72.67 ± 8.77		47.22 ± 3.12	
PreDM Value	(30.65–37.00)				
T2DM Value	(32.71–39.73)				
	Q 4	75.23 ± 6.11		51.06 ± 5.77	
PreDM Value	(37.01–48.93)				
T2DM Value	(39.74–52.86)				
	VAI				
	Q 1	73.53 ± 6.14	0.052 **	47.48 ± 4.42	0.053 **
PreDM Value	(1.05–2.73)				
T2DM Value	(1.21–3.27)				
	Q 2	70.62 ± 6.48		48.24 ± 5.71	
PreDM Value	(2.74–3.73)				
T2DM Value	(3.28–4.84)				
	Q 3	72.28 ± 4.92		49.66 ± 6.44	
PreDM Value	(3.74–5.46)				
T2DM Value	(4.85–7.57)				
	Q 4	74.54 ± 9.34		50.37 ± 4.29	
PreDM Value	(5.47–35.13)				
T2DM Value	(7.58–28.59)				

Q1: quarter 1; Q2: quarter 2; Q3: quarter 3: Q4: quarter 4; BAI: body adiposity index; VAI: visceral adiposity index; WHR: waist-to-hip ratio; WHtR: waist-to-height ratio; BMI: body mass index; * *p* < 0.05: statistically significant; **: stretched the significance limit.

**Table 6:** Comparing female and male clinical features in the PreDM and T2DM groups.

Variables (Mean ± SD) [Median (Range)]	PreDM Cohort (*n* = 50)			T2DM Cohort (*n* = 48)		
	Female	Male	*p*-Value from Pearson’s Chi-Squared/ Student’s *t*-Test	Female	Male	*p*-Value from Pearson’s Chi-Squared/ Student’s *t*-Test
Age (years) (mean ± SD)	47.90 ± 8.02	49.65 ± 7.21	0.435	70.17 ± 11.44	58.33 ± 9.37	0.0003 *
BMI category (kg/m^2^) (mean ± SD)	31.47 ± 6.27	28.84 ± 6.67	0.163	31.86 ± 5.89	30.51 ± 4.26	0.369
Normal weight (18.5–24.9 kg/m^2^)	24.50 ± 0.42	23.36 ± 0.75	0.021 *	23.26 ± 1.25	23.57 ± 0.93	0.746
Overweight (25–29.9 kg/m^2^)	27.13 ± 1.19	27.84 ± 1.65	0.273	26.08 ± 0.88	27.81 ± 1.56	0.071
Obese (≥30 kg/m^2^)	37.18 ± 4.37	38.11 ± 6.62	0.724	34.74 ± 4.31	33.78 ± 2.17	0.471
Weight (kg) (mean ± SD)	78.85 ± 16.33	89.74 ± 21.91	0.049 *	81.90 ± 16.11	93.46 ± 16.88	0.019 *
Height (cm) (mean ± SD)	168.9 ± 10.91	164.2 ± 10.78	0.140	160.3 ± 6.76	175 ± 6.80	<0.0001 *
WC (cm) (mean ± SD)	101.2 ± 15.1	102.8 ± 21.01	0.760	102.7 ± 11.49	108.9 ± 13.38	0.090
HC (cm) (mean ± SD)	109.6 ± 14.75	105 ± 9.86	0.227	109.8 ± 9.24	110.9 ± 19.47	0.814
WHR (mean ± SD)	0.92 ± 0.09	0.98 ± 0.18	0.171	0.93 ± 0.07	1.05 ± 0.49	0.257
WHtR (mean ± SD)	0.59 ± 0.08	0.62 ± 0.10	0.351	0.64 ± 0.07	0.62 ± 0.08	0.420
BAI (mean ± SD)	32.20 ± 7.76	32.13 ± 5.47	0.972	36.38 ± 6.56	30.08 ± 9.28	0.009 *
VAI [median (range)]	4.96 (1.17–35.13)	3.10 (1.05–5.57)	<0.0001 *	5.39 (2.26–28.59)	4.01 (1.21–22.26)	0.039*
AC (mean ± SD)	2.63 ± 1.42	2.90 ± 1.54	0.531	2.87 ± 1.53	3.23 ± 1.53	0.413
PNI (mean ± SD)	72.87 ± 6.97	72.42 ± 6.74	0.822	49.56 ± 6.20	48.31 ± 4.12	0.415
TC (mean ± SD)	177.5 ± 45.74	191 ± 53.51	0.347	194.3 ± 61.31	190.5 ± 61.93	0.834
TG (mg/dL) [median (range)]	111.5 (45–515)	99.5 (51–155)	0.050 *	149.5 (57–358)	125.5 (45–580)	0.483
LDL-c (mg/dL) (mean ± SD)	99.85 ± 38.82	120 ± 49.29	0.113	110 ± 43.51	101.6 ± 33.04	0.045 *
HDL-c (mg/dL) (mean ± SD)	52.16 ± 13.88	52.22 ± 14.13	0.987	51.67 ± 12.70	47.09 ± 11.47	0.196
HbA1C (%) (mean ± SD)	5.77 ± 0.50	5.82 ± 0.43	0.733	10.07 ± 2.08	9.52 ± 1.46	0.306
CKD-EPI (mL/min/1.73 m^2^) (mean ± SD)	85.96 ± 28.99	93.00 ± 25.17	0.039 *	35.89 ± 9.15	51.96 ± 10.48	<0.0001 *

* *p* < 0.05: statistically significant.

**Table 7:** Diagnostic performance of the investigated parameters.

Parameter	AUC	Std. Error	Cut-Off Values	Sensitivity %	Specificity %	Youden Index	*p*-Value
HbA1c	1.000	0.000	6.90	100.00	100.00	1.000	<0.0001
ALB	1.000	0.000	5.00	100.00	100.00	1.000	<0.0001
FPG	0.999	0.001	122.50	97.92	100.00	0.980	<0.0001
PNI	0.997	0.002	61.45	97.92	98.00	0.960	<0.0001
CKD-EPI	0.920	0.026	61.41	91.67	80.00	0.720	<0.0001
2hPG	0.911	0.032	197.50	81.25	100.00	0.810	<0.0001
AC	0.906	0.036	3.90	79.17	98.00	0.770	<0.0001
Creatinine	0.899	0.032	1.34	83.33	86.00	0.690	<0.0001
VAI	0.617	0.056	3.96	64.58	60.00	0.250	0.045
BAI	0.558	0.058	30.45	66.67	50.00	0.170	0.318
HDL-c	0.536	0.058	49.11	56.25	54.00	0.100	0.529
LDL-c	0.522	0.059	105.40	52.08	58	0.100	0.703
TC	0.529	0.059	196.50	52.08	56.00	0.080	0.618

## Data Availability

The data used to support the findings of this study are available from the corresponding author upon reasonable request. The data are not publicly available due to [ethical reasons].
